# Location of Cu^2+^ in CHA zeolite investigated by X-ray diffraction using the Rietveld/maximum entropy method

**DOI:** 10.1107/S2052252514020181

**Published:** 2014-09-23

**Authors:** Casper Welzel Andersen, Martin Bremholm, Peter Nicolai Ravnborg Vennestrøm, Anders Bank Blichfeld, Lars Fahl Lundegaard, Bo Brummerstedt Iversen

**Affiliations:** a Center for Materials Crystallography, Department of Chemistry and iNANO, Aarhus University, Langelandsgade 140, Aarhus C, DK-8000, Denmark; b Haldor Topsoe, Nymøllevej 55, Kgs. Lyngby, DK-2800, Denmark

**Keywords:** CHA zeolites, catalytic activity, location of Cu^2+^, synchrotron powder X-ray diffraction, Rietveld/maximum entropy method

## Abstract

Accurate structural models of reaction centres in zeolite catalysts are a prerequisite for mechanistic studies and further improvements to the catalytic performance. The Rietveld/maximum entropy method is applied to synchrotron powder X-ray diffraction data on fully dehydrated CHA-type zeolites with and without loading of catalytically active Cu^2+^ for the selective catalytic reduction of NO_*x*_ with NH_3_. The method identifies the known Cu^2+^ sites in the six-membered ring and a not previously observed site in the eight-membered ring. The sum of the refined Cu occupancies for these two sites matches the chemical analysis and thus all the Cu is accounted for. It is furthermore shown that approximately 80% of the Cu^2+^ is located in the new 8-ring site for an industrially relevant CHA zeolite with Si/Al = 15.5 and Cu/Al = 0.45. Density functional theory calculations are used to corroborate the positions and identity of the two Cu sites, leading to the most complete structural description of dehydrated silicoaluminate CHA loaded with catalytically active Cu^2+^ cations.

## Abstract 2

Rietveld/MEM analysis applied to synchrotron powder X-ray diffraction data of dehydrated CHA zeolites with catalytically active Cu^2+^ reveals Cu^2+^ in both the six- and eight-membered rings in the CHA framework, providing the first complete structural model that accounts for all Cu^2+^. Density functional theory calculations are used to corroborate the experimental structure and to discuss the Cu^2+^ coordination in terms of the Al distribution in the framework.

## 1. Introduction   

Efficient elimination of environmentally harmful gaseous NO_*x*_ compounds (NO, NO_2_ and N_2_O) from automotive diesel emission remains a challenging task. To meet current and future legislative demands, two different after-treatment technologies have attracted the most attention: lean NO_*x*_ traps (LNT), and selective catalytic reduction (SCR) of nitrogen oxides using ammonia, or precursors thereof, as reductant (NH_3_-SCR). Focusing on the NH_3_-SCR technology, zeolite catalysts containing transition metals have shown high activity and N_2_ selectivity (Brandenberger *et al.*, 2008 ▶; Fritz & Pitchon, 1997 ▶), and Cu-loaded zeolites in particular have attracted attention due to their high activity at temperatures below 473 K (Moden *et al.*, 2010 ▶; Colombo *et al.*, 2010 ▶).

Previous studies have focused on Cu^2+^ ion-exchanged MFI zeolites (Cu-ZSM-5), Cu^2+^ ion-exchanged *BEA zeolites (Cu-Beta) and Cu^2+^ ion-exchanged FAU zeolites (Cu-Y) (Iwamoto *et al.*, 1986 ▶, 1981 ▶; Brandenberger *et al.*, 2008 ▶). Zeolite-based catalysts are favoured over vanadia-based systems due to their hydrothermal stability, but stability remains an issue for practical applications where high-temperature excursions can occur (*e.g.* ≥ 923 K). Although Cu-Beta shows improved hydrothermal stability compared with Cu-ZSM-5 (Kwak, Tran *et al.*, 2012 ▶), further improvement of the durability is required by the automotive industry in order to meet legislative demands (Johnson, 2011 ▶). A leap forward in terms of both stability and selectivity has been offered by the use of small-pore zeolites (Kwak *et al.*, 2010 ▶; Fickel *et al.*, 2011 ▶; Blakeman *et al.*, 2014 ▶), where framework dealumination and migration are suppressed. Most promising are zeolites and zeotypes with the chabazite (CHA) framework, such as Cu-SSZ-13 and Cu-SAPO-34, which have both been commercialized as DeNOx catalysts for diesel-powered automotive applications (Gao *et al.*, 2013 ▶; Kwak *et al.*, 2010 ▶; Fickel & Lobo, 2009 ▶; Blakeman *et al.*, 2014 ▶). Despite this successful commercialization, many details of the zeolite SCR mechanism are poorly understood. Detailed structural models of the catalysts are prerequisites for an in-depth understanding of and further improvements in performance. Because of its simple structure and limited number of cation exchange positions, the CHA framework topology offers an unprecedented opportunity to study a highly commercially relevant catalyst in great detail as if it were a model system.

Properties like valence state, local atomic coordination, concentration and position of the Cu species within the framework structure are all crucial parameters for understanding their role in SCR catalysis. Hence, several studies have been dedicated to determining these properties using mainly spectroscopic techniques (Kwak, Zhu *et al.*, 2012 ▶; Zamadics *et al.*, 1992 ▶; Gao *et al.*, 2013 ▶; Wang *et al.*, 2014 ▶; Xue *et al.*, 2013 ▶; Deka *et al.*, 2013 ▶). A few studies also used structural modelling of diffraction data (Deka *et al.*, 2012 ▶; Calligaris & Nardin, 1982 ▶; Pluth *et al.*, 1977 ▶). Here, we apply a combination of synchrotron powder X-ray diffraction (PXRD) and the Rietveld/maximum entropy method (MEM), an approach that has proved to be sensitive towards low atomic site occupancies while maintaining a complete structural description (Takata *et al.*, 2001 ▶). The main objective of the Rietveld/MEM analysis is to improve the structural model obtained by Rietveld analysis through iterative MEM electron-density distribution (EDD) calculations. The strength of this approach lies in the model-independent MEM, which provides the possibility of visually and directly locating non-framework species, such as the cations in zeolites, based only on observed structure factors.

Cu zeolites are very dynamic systems, and the local structure and the valence state of the Cu species are sensitive to variations in pressure, temperature and composition of the surrounding atmosphere (Giordanino *et al.*, 2013 ▶). In the present study, it is ensured that the pretreatment (O_2_ activation) leaves the Cu-CHA sample in a well defined catalytically relevant dehydrated state, with dispersed monomeric Cu^2+^ species (Giordanino *et al.*, 2013 ▶). The current structural model of Cu-CHA is based on the model by Fickel & Lobo (2009 ▶). The sample studied by Fickel and Lobo had been heated in an inert atmosphere to temperatures that are now known to reduce Cu^2+^ to Cu^1+^ (Giordanino *et al.*, 2013 ▶). In the recent studies by Deka *et al.* (2012 ▶, 2013 ▶), the samples were pretreated in an oxidizing atmosphere and XANES data confirmed that Cu was present as Cu^2+^. Their structure model, which is based on the structure by Fickel and Lobo, identifies only 25% of the Cu in the 6-ring. The remaining 75% of the Cu known to be present from chemical analysis is not accounted for by the structure model. The motivation for this study is to obtain a complete structure model for Cu^2+^-CHA.

## 2. Experimental   

For this study, a CHA-type silicoaluminate zeolite (Si/Al = 15.5) was synthesized using the fluoride route to minimize the number of structural defects. The synthesis procedure is based on the known literature (Diaz-Cabanas & Barrett, 1998 ▶; Eilertsen *et al.*, 2012 ▶). A protonated form (H-CHA) was obtained directly after calcination and Cu-CHA (Cu/Al = 0.45) was prepared by Cu^2+^ aqueous ion-exchange followed by calcination. To dehydrate the samples for PXRD measurements, the samples were loaded into glass capillaries, then slowly heated in air and maintained at 573 K for 1 h before sealing the capillary (see Fig. S1). High-resolution synchrotron PXRD data were measured at SPring8 in Japan, beamline BL44B2, λ = 0.499818 (3) Å. Rietveld refinements were performed using the sin θ/λ range 0.03–0.77 Å^−1^ and the software *JANA2006* (Petříček *et al.*, 2006 ▶). Bérar-Baldinozzi asymmetry was applied (Bérar & Baldinozzi, 1993 ▶). The occupancies of Si, Al and O were kept fixed for all refinements: 33.8 Si per unit cell, 2.2 Al per unit cell and 72.0 O per unit cell. Rietveld-refined models were visualized using *DIAMOND* (Putz & Brandenburg, 2012 ▶). MEM calculations were performed with the Sakato–Sato algorithm using *BayMEM* (van Smaalen *et al.*, 2003 ▶; Sakata & Sato, 1990 ▶). MEM EDDs were visualized using *VESTA* (Momma & Izumi, 2011 ▶). Density functional theory (DFT) calculations were performed using a real-space grid-based projector augmented-wave method (GPAW) (Mortensen *et al.*, 2005 ▶; Enkovaara *et al.*, 2010 ▶) interfaced using the atomic simulation environment (ASE) (Bahn & Jacobsen, 2002 ▶).

## 3. Results and discussion   

### 3.1. H-CHA   

**Figure 1:**
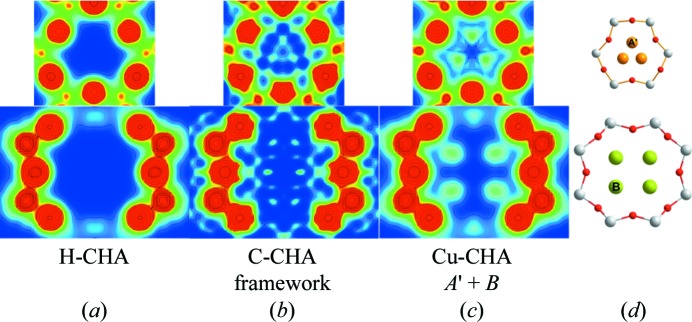
Two-dimensional MEM EDDs of the 6R (top) and 8R (bottom), going from 0 e Å^−2^ (blue) to 1 e Å^−2^ (red), of (*a*) H-CHA, (*b*) Cu-CHA using only the framework as an MEM prior, and (*c*) Cu-CHA using the improved structure factors obtained from Rietveld analysis including the *A*′ and *B* sites in the structure model. (*d*) Ball-and-stick drawings of the 6R (top) and 8R (bottom) of Cu-CHA, showing the Cu^2+^ sites *A*′ (orange) and *B* (green), and the *T* sites (Si/Al, white) oxygen (red).

First, the H-CHA zeolite was investigated using the Rietveld/MEM analysis method. Well established structural parameters for the framework were taken from the IZA structure database (IZA-SC, 2007 ▶) and used in the initial Rietveld structure model. Atomic, thermal and profile parameters were refined. From the final Rietveld refinement, the background-corrected observed structure factors were extracted for MEM in accordance with the independent spherical atom model (ISAM). The refined structural model was used as a procrystal density (MEM prior) for the MEM analysis. The MEM prior is the initial MEM EDD, which is perturbed by the observed structure factors in the MEM optimization. Residual density analysis (RDA) was used as a basis for optimized stopping criteria in the MEM calculations (Bindzus & Iversen, 2012 ▶; Meindl & Henn, 2008 ▶). The final MEM EDDs of the six-membered ring (6R) and eight-membered ring (8R) in H-CHA are shown in Fig. 1 ▶(*a*). The EDD shows no noise down to an isosurface level of electron density (ED) below 0.05 e Å^−3^, which is a clear indication of excellent sample and data quality. Additionally, there is no trace of extra density in the zeolite voids, which provides evidence of high-purity samples and an effective dehydration method. Thus, H-CHA provides an excellent reference model and gives an indication of the expected noise level for similar metal-loaded CHA zeolites.

### 3.2. Cu-CHA   

#### 3.2.1. Rietveld/MEM   

Turning to Cu-CHA, the refined structure model of H-CHA was used in the initial Rietveld refinement. The following MEM analysis leads to the EDDs shown in Fig. 1 ▶(*b*). Comparing with H-CHA, it is clear that ED is observed in several non-framework locations with varying intensity (see also Fig. S15). The largest occurrences of non-framework ED are located mainly within the 6R and the 8R. Inspection of the ED in the 6R reveals a Cu site (*A*′) shifted slightly away from the central symmetry axis towards the framework oxygen shared by the 8R and 6R (atom O3; see Table S1). This site is in agreement with the most recent structure model of Cu-CHA by Deka *et al.* (2012 ▶). The ED observed in the 8R suggests that Cu is also located here, but the exact location is less clear. A series of structure models, each containing the *A*′ site and one of the MEM maxima observed close to the 8R, was tested using Rietveld analysis. The multiple Rietveld refinements, where the atomic positions and occupancies of the Cu^2+^ cations were refined, revealed a new stable site (*B*) with high occupancy in the plane of the 8R (see Fig. 1 ▶ *d*). Even after removal of the *A*′ site from the structure model, the position and occupancy of the *B* site remain unchanged and stable. The final Rietveld model parameters can be found in Table 1 ▶ and further details are given in Table S1. MEM analysis based on the observed structure factors obtained with the new structure model and using the model as an MEM prior results in an EDD with clear maxima at the *A*′ and *B* sites (Fig. 1 ▶ *c*). The smooth contour lines implying spherical atoms, the low noise level and the fact that no additional MEM maxima are observed confirm that a complete structure model has been obtained. Furthermore, the fact that the total amount of Cu in the refined structure model [1.03 (5) Cu per unit cell] is very close to that expected from chemical analysis [0.98 (3) Cu per unit cell] supports the validity of the new structure model. The existence of an extra site beyond the 6R site has been proposed several times (Kwak, Zhu *et al.*, 2012 ▶; Deka *et al.*, 2012 ▶), but its exact location has not been shown experimentally before now.

There is some uncertainty in the literature regarding the exact position of Cu in the 6R. In addition to the identified *A*′ site, another site (*A*) centred in the 6R on the symmetry axis has also been proposed (Pluth *et al.*, 1977 ▶; Fickel & Lobo, 2009 ▶). A low Cu occupancy, combined with large correlations between the occupancies and the thermal and atomic position parameters of the closely spaced *A* and *A*′ sites, makes it difficult to determine their relative occupancies definitively based on PXRD analysis alone. However, to explore the possibility of an occupied *A* site, Rietveld/MEM was performed on a structure model including only the *A* and *B* sites. The Cu position from Fickel & Lobo (2009 ▶) was taken as an initial guess for the *A* site. The final refinement reached the same reliability factors, with similar occupancies (see Table 1 ▶), as the refinement including only the *A*′ and *B* sites. The thermal parameter for site *A* is larger than that for site *A*′, and most notably the *B* site is virtually unchanged and stable.

#### 3.2.2. DFT   

To obtain further insight concerning the identity and exact positions of the different Cu species, DFT was applied (computational details and methods are given in the supporting information). Since Si/Al = 15.5, the number of Al atoms in the unit cell is, on average, approximately two. Although the Al distribution in zeolites cannot be considered to be random (Dědeček *et al.*, 2012 ▶). As pointed out by Bates *et al.* (2014 ▶), a certain fraction of these Al pairs will be in the 6R, whereas many of the remaining sites will be related to isolated framework Al atoms.

**Figure 2:**
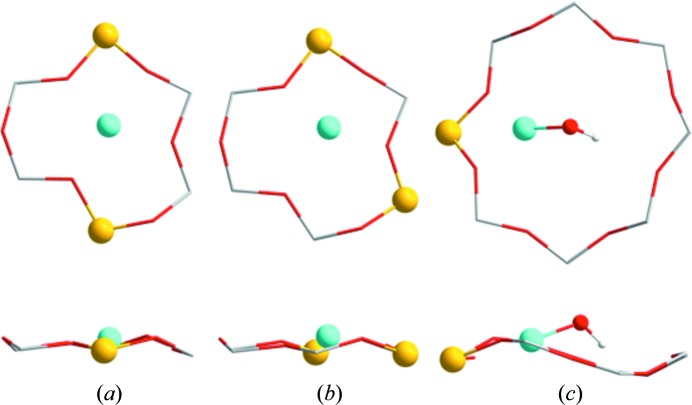
DFT calculations of (*a*) the 6R with two Al atoms (yellow) located diagonally and a Cu^2+^ cation in the centre; (*b*) the 6R with two Al atoms sitting as next-nearest-neighbours and a Cu^2+^ cation slightly shifted away from the centre; and (*c*) the 8R with a single Al atom and a [Cu^2+^(OH)]^+^ complex in the plane of the ring. (Top) Plane views and (bottom) side views of the rings.

To assess all possible Al pairs, every combination of isomorphous substitution of two Si with two Al separated by either one or two Si atoms was explored, together with the insertion of a Cu atom. In this way, all configurations with bare Cu cations that correspond to Cu^2+^ were probed, which was confirmed by the converged non-zero magnetic moment of the Cu atom in the calculations. From these systematic calculations, two specific configurations were found to be more stable by more than 50 kJ mol^−1^ compared with all other optimized configurations (see Figs. 2 ▶ *a* and 2 ▶ *b*). These configurations are characterized by having two Al atoms in the same 6R, either diagonally across (Fig. 2 ▶ *a*) or separated by a single Si atom (Fig. 2 ▶ *b*). Depending on the location of the Al atoms in the 6R, various degrees of shift away from the centre of the 6R are seen for the Cu location. Consequently, these two configurations are interpreted as sites *A* and *A*′, respectively.

Secondly, isomorphous substitution of a single Al atom with Si reveals the most stable Cu site to be in the 6R. However, the magnetic moment converged to zero, indicating a Cu^+^ cation. This site corresponds well with the current model of Fickel & Lobo (2009 ▶). A Cu complex including an OH ligand has recently been suggested, based on spectroscopic observations on O_2_-activated Cu-CHA (Giordanino *et al.*, 2013 ▶). Introduction of an OH ligand revealed the most stable Cu site to be in the 8R as a [Cu^II^(OH)]^+^ complex (see Fig. 2 ▶ *c*). This specific Cu cation resembles the Cu^2+^ confirmed by the converged non-zero magnetic moment. This particular complex is slightly stabilized by hydrogen bonding between the H atom in the complex and a framework O atom located diagonally across the 8R. Perhaps most important for our investigation is that it was not possible to make any stable configurations with a [Cu^II^(OH)]^+^ complex in the plane of the 6R. For this reason, we assign the [Cu^II^(OH)]^+^ complex to be the Cu species at the *B* site found by Rietveld/MEM. Furthermore, as the preferred location of this complex is in the 8R, and it is only accompanied by a small energy difference and barrier when shifted out of the 8R plane (Fig. 2 ▶ *c*), large displacement parameters can be expected for this specific site, as indeed observed by the Rietveld/MEM results (see *U*_iso_ in Table 1 ▶, and Fig. 1 ▶ *c*).

In combination, the Rietveld/MEM and DFT results provide strong evidence for Cu^2+^ cations being located in at least two well defined positions related to the 6R and 8R. The positions have been accurately determined using Rietveld/MEM, while DFT calculations determined a plausible cause of site preference in the 6R based on Al distribution. The DFT results also suggested a [Cu^II^(OH)]^+^ complex to be the Cu species in the 8R.

#### 3.2.3. Summary   

**Figure 3:**
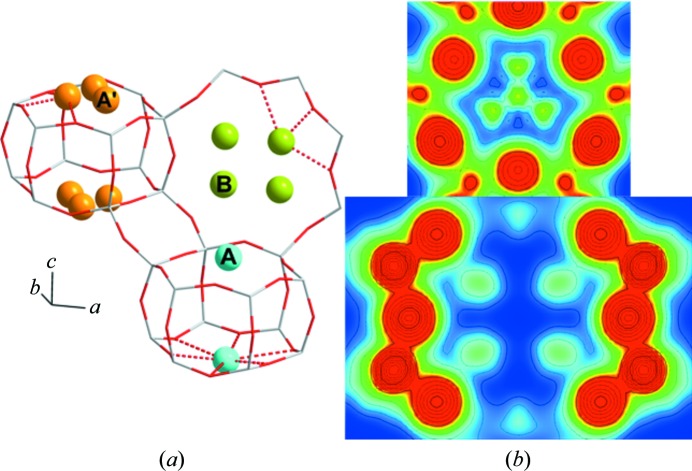
(*a*) The final model of Cu-CHA, showing the Cu^2+^ sites *A* (cyan), *A*′ (orange) and *B* (green). (*b*) Two-dimensional MEM EDDs of the 6R (top) and 8R (bottom) of Cu-CHA, going from 0 e Å^−2^ (blue) to 1 e Å^−2^ (red).

Taking the collected results from Rietveld/MEM and DFT into account, a final Rietveld/MEM analysis including all three sites *A, A*′ and *B* was performed. In order to overcome the large atomic parameter correlation of the 6R sites, the displacement parameters of sites *A* and *A*′ were refined as one. Furthermore, the atomic positions for the two 6R sites were fixed to the positions retrieved from the above-mentioned Rietveld refinements given in Table 1 ▶. The resulting MEM EDD displays a propeller-shaped ED, with relative occupancies of *A*′/*A* ≃ 3. This final Rietveld/MEM analysis cements the *B* site in the 8R as a new stable Cu^2+^ site in Cu-CHA; the parameters are, on the whole, unchanged (see Table 1 ▶ and Fig. 3 ▶).

## 4. Conclusions   

In conclusion, we find that the Cu^2+^ cations in Cu-CHA are not only located in the 6R, but a significant proportion are located at a specific crystallographic site in the 8R (∼80% of the Cu). This finding provides the first unambiguous experimental proof and localization of the Cu^2+^ site in the 8R. In contrast with previous structure models, this new model fully describes a Cu^2+^-CHA zeolite, accounting for all Cu found to be present by elemental analysis. This structure model not only provides structural insight, which is essential for a detailed understanding of the Cu zeolite NH_3_-SCR mechanism, it also provides a useful tool for further studies of the Cu distribution between these sites when varying Cu load and Si/Al ratio.

## Supplementary Material

Extra figures and tables. DOI: 10.1107/S2052252514020181/fc5004sup1.pdf

## Figures and Tables

**Table 1:** Relative atomic coordinates and occupancies (Occ) for the Cu^2+^ sites, including the reliability factors *wR*_p_ and *R*_p_, obtained by Rietveld/MEM analysis of synchrotron PXRD data; *U*_iso_ is given in ^2^

Site		*A* + *B*	*A* + *B*	*A* + *A* + *B*
*A*	*z*/*c*		0.147(5)	0.147†
6*c*	*U*_iso_		0.11(5)	0.01(3)‡
*x* = *y* = 0	Occ§		0.025(3)/0.15(2)	0.008(2)/0.05(1)
*A*	*x*/*a*	0.040(3)		0.039†
18*h*	*z*/*c*	0.150(4)		0.150†
*y* = 2*x*	*U*_iso_	0.10(5)		0.01(3)‡
	Occ§	0.013(1)/0.22(2)		0.008(1)/0.14(2)
*B*	*x*/*a*	0.997(3)	0.996(4)	0.997(3)
36*i*	*y*/*b*	0.413(3)	0.413(3)	0.412(3)
	*z*/*c*	0.069(2)	0.068(3)	0.068(2)
	*U*_iso_	0.16(3)	0.16(3)	0.16(3)
	Occ§	0.022(1)/0.80(4)	0.021(1)/0.76(4)	0.022(1)/0.79(4)
Occ per unit cell		1.03(5)	0.91(4)	0.98(5)
*wR*_p_		0.0267	0.0268	0.0267
*R*_p_		0.0199	0.0198	0.0198

† Values not refined.

‡ Refined as one parameter.

§ Fractional site occupancy/number of atoms per unit cell.

## References

[bb1] Bahn, S. R. & Jacobsen, K. W. (2002). *Comput. Sci. Eng.* **4**, 56–66.

[bb2] Bates, S. A., Verma, A. A., Paolucci, C., Parekh, A. A., Anggara, T., Yezerets, A., Schneider, W. F., Miller, J. T., Delgass, W. N. & Ribeiro, F. H. (2014). *J. Catal.* **312**, 87–97.

[bb38] Bérar, J.-F. & Baldinozzi, G. (1993). *J. Appl. Cryst.* **26**, 128–129.

[bb3] Bindzus, N. & Iversen, B. B. (2012). *Acta Cryst.* A**68**, 750–762. 10.1107/S0108767312037269 23075617

[bb4] Blakeman, P. G., Burkholder, E. M., Chen, H.-Y., Collier, J. E., Fedeyko, J. M., Jobson, H. & Rajaram, R. R. (2014). *Catal. Today*, **231**, 56–63.

[bb5] Brandenberger, S., Kröcher, O., Tissler, A. & Althoff, R. (2008). *Catal. Rev.* **50**, 492–531.

[bb6] Calligaris, M. & Nardin, G. (1982). *Zeolites*, **2**, 200–204.

[bb7] Colombo, M., Nova, I. & Tronconi, E. (2010). *Catal. Today*, **151**, 223–230.

[bb8] Dědeček, J., Sobalík, Z. & Wichterlová, B. (2012). *Catal. Rev.* **54**, 135–223.

[bb9] Deka, U., Juhin, A., Eilertsen, E. A., Emerich, H., Green, M. A., Korhonen, S. T., Weckhuysen, B. M. & Beale, A. M. (2012). *J. Phys. Chem. C*, **116**, 4809–4818.

[bb10] Deka, U., Lezcano-Gonzalez, I., Warrender, S. J., Lorena Picone, A., Wright, P. A., Weckhuysen, B. M. & Beale, A. M. (2013). *Microporous Mesoporous Mater.* **166**, 144–152.

[bb11] Diaz-Cabanas, M.-J. & Barrett, P. A. (1998). *Chem. Commun.* **17**, 1881–1882.

[bb12] Eilertsen, E. A., Arstad, B., Svelle, S. & Lillerud, K. P. (2012). *Microporous Mesoporous Mater.* **153**, 94–99.

[bb13] Enkovaara, J. *et al.* (2010). *J. Phys. Condens. Matter*, **22**, 253202. 10.1088/0953-8984/22/25/253202 21393795

[bb14] Fickel, D. W., D’Addio, E., Lauterbach, J. A. & Lobo, R. F. (2011). *Appl. Catal. B Environ.* **102**, 441–448.

[bb15] Fickel, D. W. & Lobo, R. F. (2009). *J. Phys. Chem. C*, **114**, 1633–1640.

[bb16] Fritz, A. & Pitchon, V. (1997). *Appl. Catal. B Environ.* **13**, 1–25.

[bb17] Gao, F., Walter, E. D., Karp, E. M., Luo, J., Tonkyn, R. G., Kwak, J. H., Szanyi, J. & Peden, C. H. F. (2013). *J. Catal.* **300**, 20–29.

[bb18] Giordanino, F., Vennestrøm, P. N. R., Lundegaard, L. F., Stappen, F. N., Mossin, S., Beato, P., Bordiga, S. & Lamberti, C. (2013). *Dalton Trans.* **42**, 12741–12761. 10.1039/c3dt50732g 23842567

[bb19] Iwamoto, M., Furukawa, H., Mine, Y., Uemura, F., Mikuriya, S. & Kagawa, S. (1986). *J. Chem. Soc. Chem. Commun.* pp. 1272–1273.

[bb20] Iwamoto, M., Yokoo, S., Sakai, K. & Kagawa, S. (1981). *J. Chem. Soc. Farad. Trans. 1*, **77**, 1629–1638.

[bb21] IZA-SC (2007). *Database of Zeolite Structures.* http://www.iza-structure.org/databases/.

[bb22] Johnson, T. V. (2011). *SAE Int. J. Engines*, **4**, 143–157.

[bb23] Kwak, J. H., Tonkyn, R. G., Kim, D. H., Szanyi, J. & Peden, C. H. F. (2010). *J. Catal.* **275**, 187–190.

[bb24] Kwak, J. H., Tran, D., Burton, S. D., Szanyi, J., Lee, J. H. & Peden, C. H. F. (2012). *J. Catal.* **287**, 203–209.

[bb25] Kwak, J. H., Zhu, H., Lee, J. H., Peden, C. H. F. & Szanyi, J. (2012). *Chem. Commun.* **48**, 4758–4760. 10.1039/c2cc31184d 22473309

[bb39] Meindl, K. & Henn, J. (2008). *Acta Cryst.* A**64**, 404–418. 10.1107/S0108767308006879 18421130

[bb26] Moden, B., Donohue, J. M., Cormier, W. E. & Li, H.-X. (2010). *Top. Catal.* **53**, 1367–1373.

[bb27] Momma, K. & Izumi, F. (2011). *J. Appl. Cryst.* **44**, 1272–1276.

[bb28] Mortensen, J. J., Hansen, L. B. & Jacobsen, K. W. (2005). *Phys. Rev. B*, **71**, 035109.

[bb29] Petříček, V., Dušek, M. & Palatinus, L. (2006). *JANA2006.* Institute of Physics, Czech Academy of Sciences, Prague, Czech Republic.

[bb30] Pluth, J. J., Smith, J. V. & Mortier, W. J. (1977). *Mater. Res. Bull.* **12**, 1001–1007.

[bb31] Putz, H. & Brandenburg, K. (2012). *DIAMOND.* Crystal Impact GbR, Bonn, Germany.

[bb32] Sakata, M. & Sato, M. (1990). *Acta Cryst.* A**46**, 263–270.

[bb33] Smaalen, S. van, Palatinus, L. & Schneider, M. (2003). *Acta Cryst.* A**59**, 459–469. 10.1107/S010876730301434X 12944610

[bb34] Takata, M., Nishibori, E. & Sakata, M. (2001). *Z. Kristallogr.* **216**, 71–86.

[bb35] Wang, D., Zhang, L., Li, J., Kamasamudram, K. & Epling, W. S. (2014). *Catal. Today*, **231**, 64–74.

[bb36] Xue, J., Wang, X., Qi, G., Wang, J., Shen, M. & Li, W. (2013). *J. Catal.* **297**, 56–64.

[bb37] Zamadics, M., Chen, X. & Kevan, L. (1992). *J. Phys. Chem.* **96**, 2652–2657.

