# Supplementary material for: Location of Cu2+ in CHA zeolite investigated by X-ray diffraction using the Rietveld/maximum entropy method
Source: IUCrJ. 2014 Sep 23;1(Pt 6):382–6. doi: 10.1107/S2052252514020181 (PMC4224456; doi:10.1107/S2052252514020181)
Supplement: Supplementary file 1 [file m-01-00382-sup1.pdf]

IUCrJ

Volume 1 (2014)

Supporting information for article:

Location of Cu^{2+} in CHA zeolite investigated by X-ray diffraction using the Rietveld/maximum entropy method

Casper Welzel Andersen, Martin Bremholm, Peter Nicolai Ravnborg Vennestrøm, Anders Bank Blichfeld, Lars Fahl Lundegaard and Bo Brummerstedt Iversen

S1. Materials preparation

The CHA zeolite was prepared from a reaction synthesis gel with the following composition:

$$1.0\ SiO_2 : 0.033\ Al_2O_3 : 0.5\ TMAdaOH : 0.5\ HF : 3\ H_2O$$

in a method very similar to the one reported earlier where TMAdaOH is the template N,N,N-trimethyladamantammonium hydroxide (Diaz-Cabanas & A. Barrett, 1998, Eilertsen *et al.*, 2012). The same preparation was also used in the study reported by Giordanino *et al.* (2013) For the specific synthesis, a gel was made using aluminum isopropoxide (> 98 %, Aldrich), tetraethyl orthosilicate (> 99 %, Aldrich), template solution (25 wt. %, Sacchem INC), and hydrofluoric acid (48 wt. %, > 99.99 %, Sigma-Aldrich). To obtain the CHA zeolite the gel was crystallized for 3 days at 150°C in autoclaves under rotation (approx. 30 rpm). The template was removed by calcination at 580°C for 3 hours in air and the copper was then introduced by aqueous Cu^{2+} ion-exchange using copper(II)acetate and further calcined at 500°C in air for 3 hours in order to remove residual acetate ligands. The expected Si/Al-ratio is approx. 15. From chemical analysis the Si/Al-ratio is found to be 15.5(8).

Zeolite powders were loaded into glass capillaries and dehydrated in air for 1 hour by a slow ramp to 300°C and sealed at the same temperature. The result from the dehydration technique can be seen in Figure S1, where the dehydration of Cu-SSZ-13 (Cu-CHA), Cu-ZSM-5, and Cu-BEA samples are shown before and after. From this a qualitative check may now be performed based on coloration to see whether the sample is dehydrated or not.

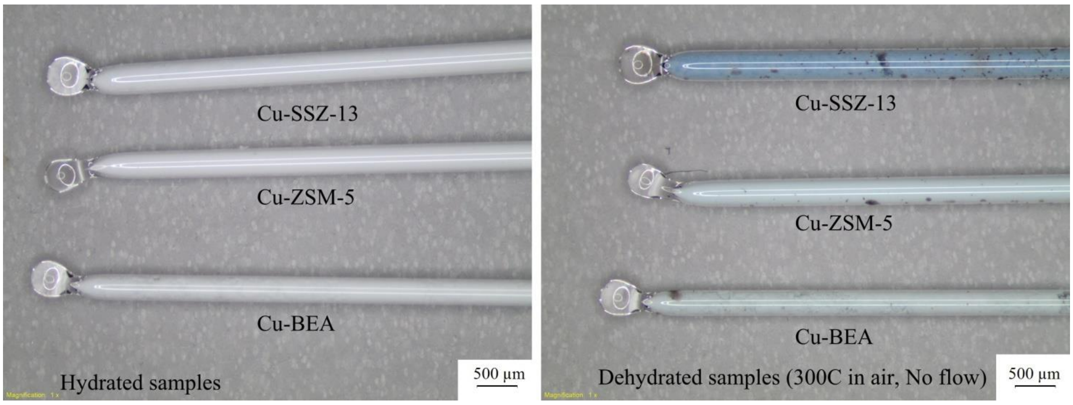

Figure S1 Pictures of different Cu ion-exchanged zeolite samples hydrated (left) and dehydrated (right). Notice the blue color of the dehydrated samples, especially the Cu-SSZ-13 (Cu-CHA) zeolite.

S2. Rietveld refinements

The chosen unit cell is hexagonal with space group $R\bar{3}m$. Furthermore, the occupancies of all sites are fixed. For the T site (Si/Al) the occupancy was calculated using the relative concentration Si/Al = 15.5(8). Isotropic thermal vibration parameters, U_{iso}, for the oxygen atoms were refined as a single parameter. It was attempted to refine all sites using anisotropic thermal vibration, but the refinement only became physically correct if the anisotropy was limited to the T site. The Lorentzian peak profile parameter Y was fixed to 0. Furthermore, Bérar-Baldinozzi asymmetry was applied (Berar & Baldinozzi, 1993).

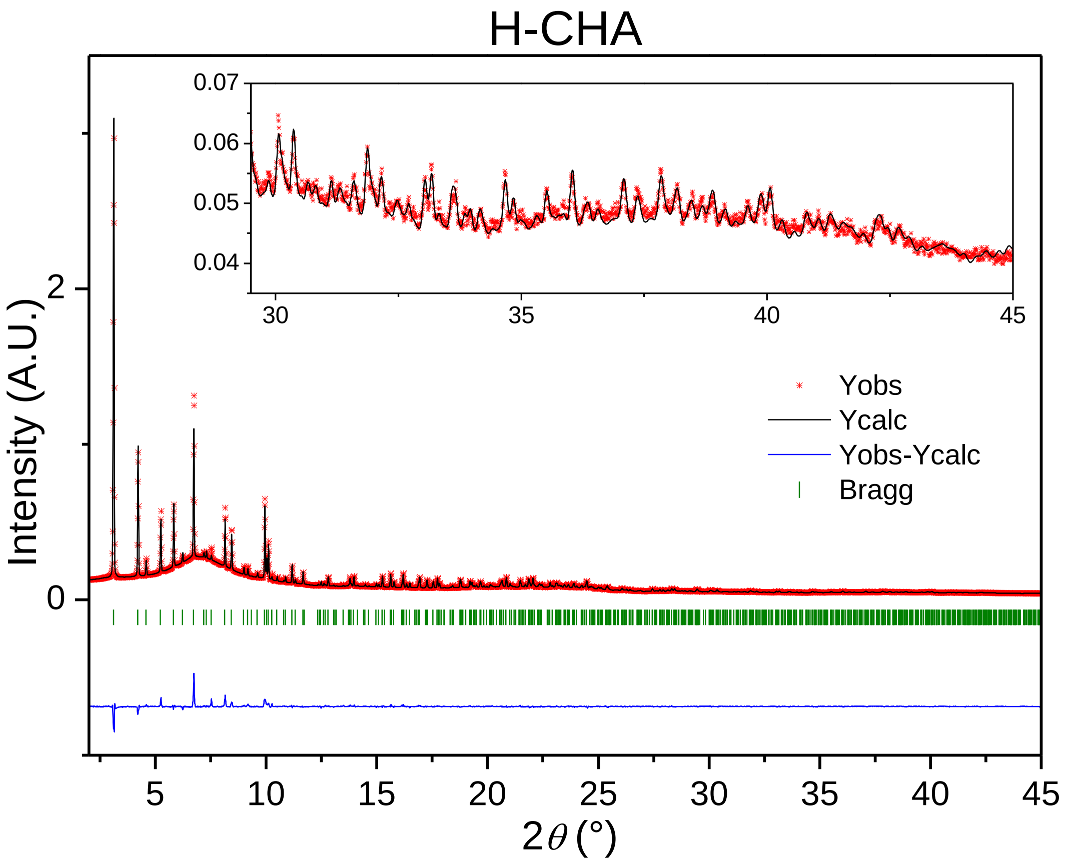

Figure S2 Rietveld refinement of H-CHA.

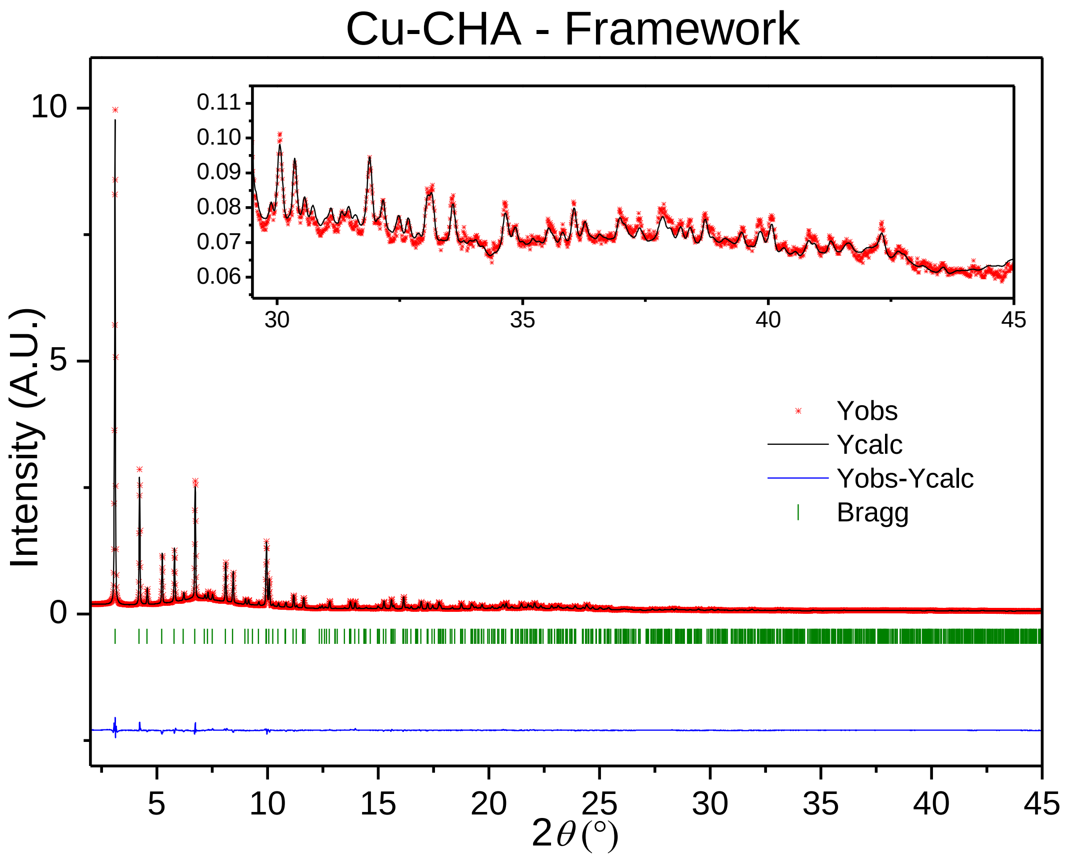

Figure S3 Rietveld refinement of Cu-CHA using only the framework.

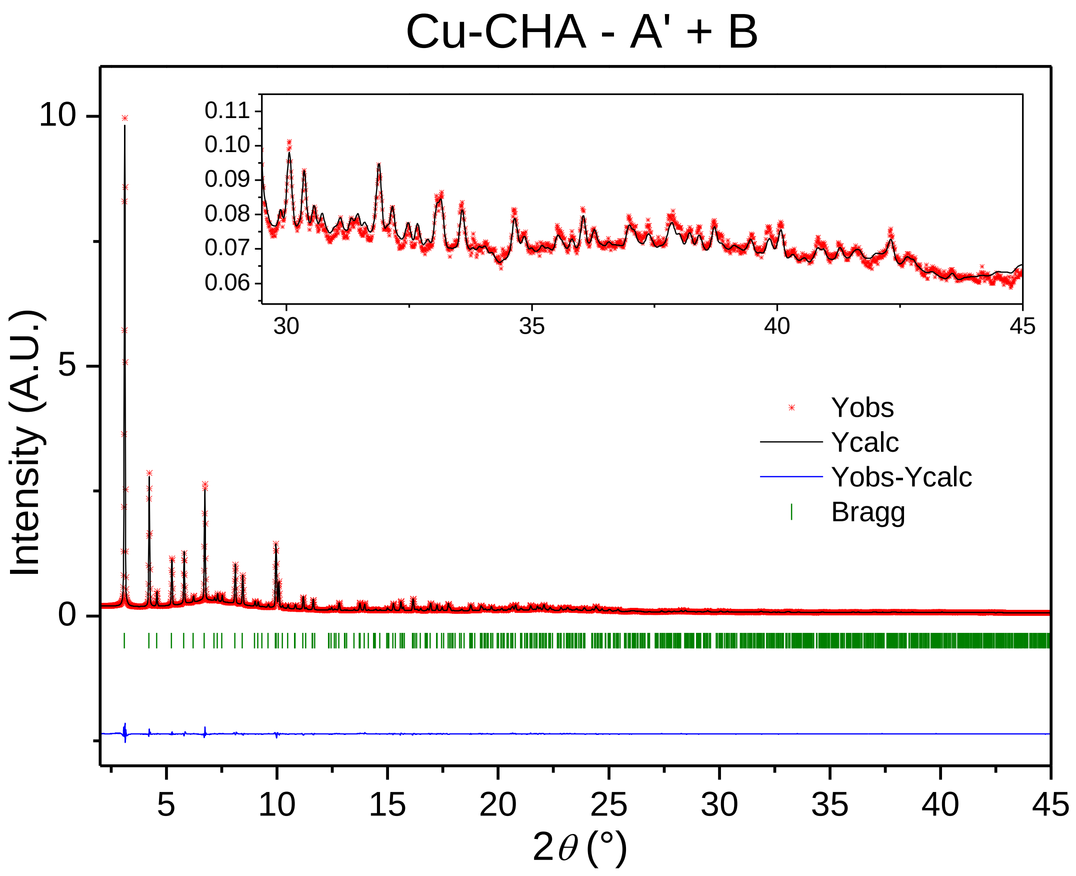

Figure S4 Rietveld refinement of Cu-CHA using the framework and Cu^{2+} sites A' and B.

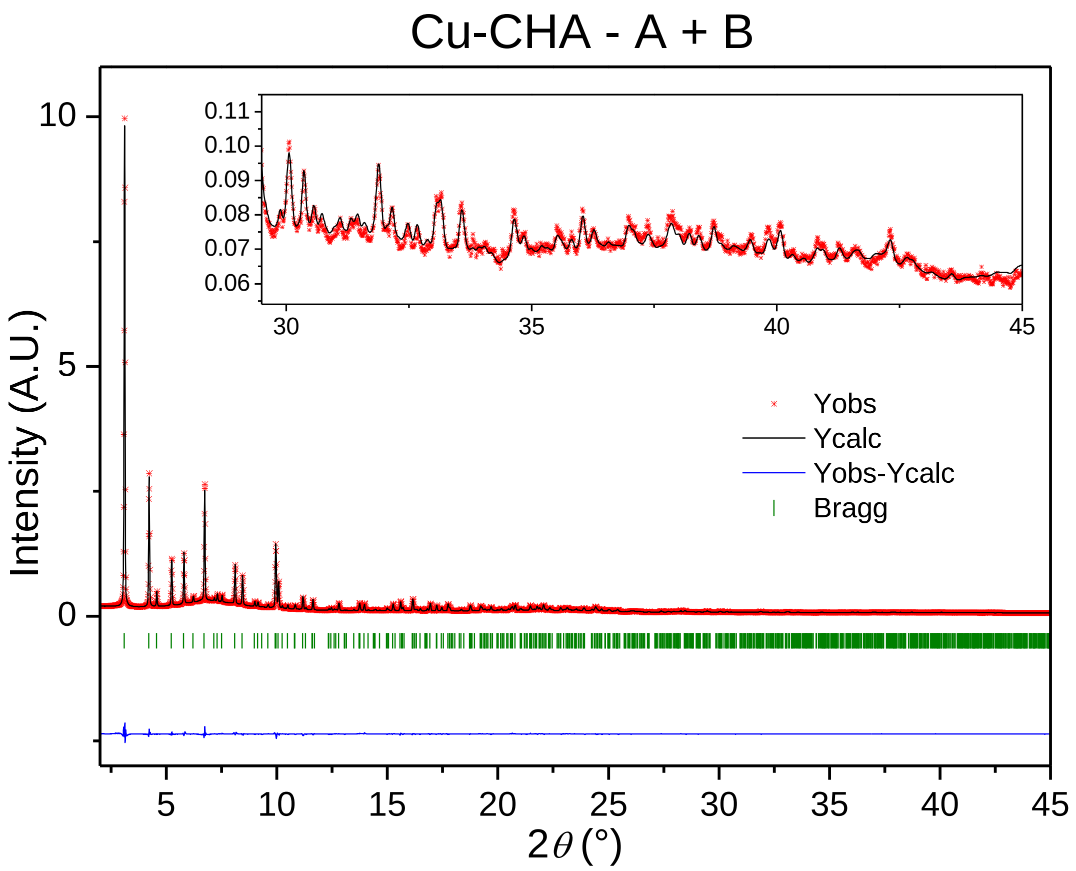

Figure S5 Rietveld refinement of Cu-CHA using the framework and Cu^{2+} sites A and B.

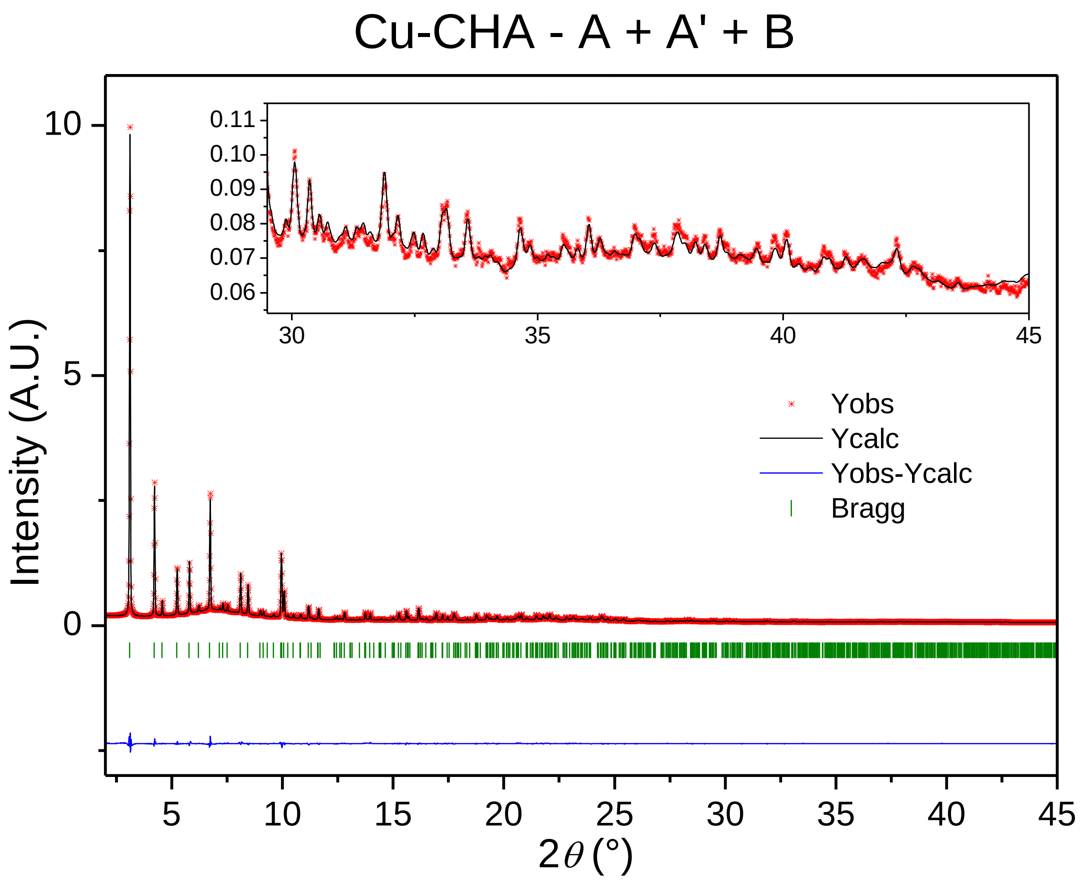

Figure S6 Rietveld refinement of Cu-CHA using the framework and Cu^{2+} sites A, A', and B.

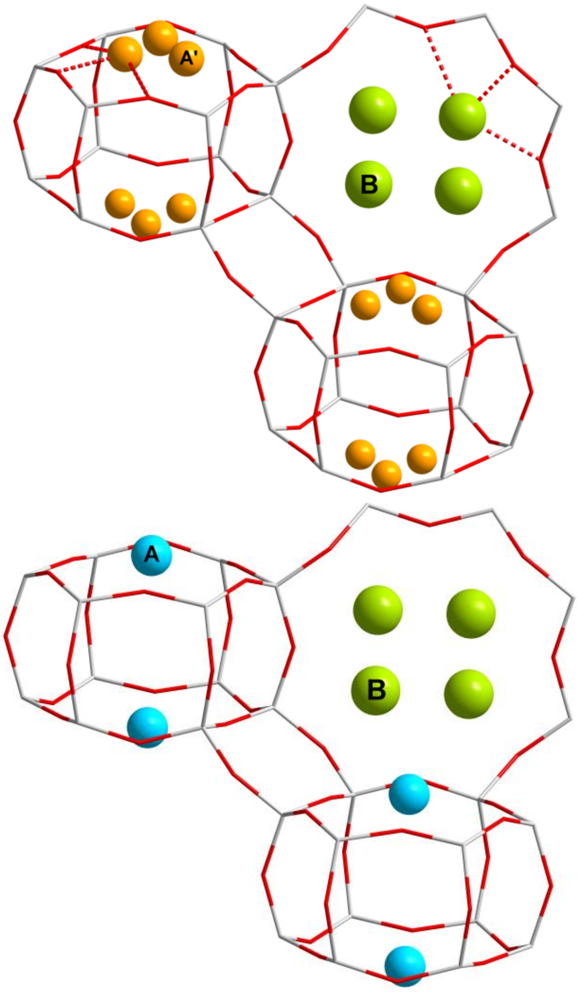

Figure S7 Structural models of Cu-CHA using the framework and Cu^{2+} sites B (lime) and (left) A' (orange) or (right) A (cyan).

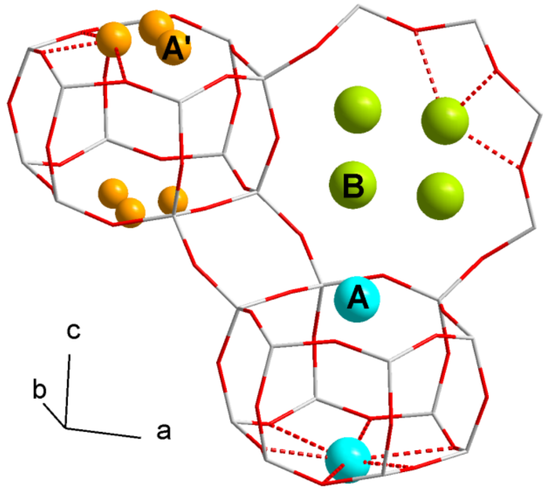

Figure S8 Structural model of Cu-CHA using the framework and Cu^{2+} sites A (cyan), A' (orange), and B (lime). Notice sites A and A' have been depicted in separate double 6-rings.

Table S1 Final Rietveld refinements of synchrotron PXRD data with λ = 0.49982 Å, 2θ range 2° to 45°, and space group $R\bar{3}m$. Occupation (fractional/no. per unit cell). Si/unit cell = 33.8. Al/unit cell = 2.2. O/unit cell = 72.0. Oxygen U_{iso}'s are refined as a single parameter. Crystallographic Information File (CIF) is also available online.

CHA		H	Cu			
Site			Framework	A' + B	A + B	A + A' + B
T (Si/Al)	x	0.0038(3)	0.0010(1)	0.0002(1)	0.0002(1)	0.0002(1)
36i	y	0.2302(2)	0.2292(1)	0.2290(1)	0.2288(1)	0.2289(1)
	z	0.1047(1)	0.10366(8)	0.10348(8)	0.10357(8)	0.10350(8)
	U_{11}	0.0005(14)	0.0165(9)	0.0163(9)	0.0163(9)	0.0164(9)
	U_{22}	0.0024(17)	0.0146(11)	0.0180(10)	0.0175(10)	0.0180(10)
	U_{33}	0.0050(11)	0.0230(8)	0.0223(7)	0.0223(7)	0.0221(7)
	U_{12}	0.0003(16)	0.0044(11)	0.0090(10)	0.0080(10)	0.0089(10)
	U_{13}	0.0031(15)	–0.0061(11)	–0.0051(10)	–0.0046(10)	–0.0051(10)
	U_{23}	–0.0028(15)	–0.0028(11)	–0.0046(10)	–0.0042(10)	–0.0045(10)
O1	x	0.8999(2)	0.9016(1)	0.9009(2)	0.9010(2)	0.9010(1)
18h	z	0.1220(4)	0.1193(3)	0.1191(2)	0.1190(2)	0.1190(2)
y = 1–x	U_{iso}	0.0062(10)	0.0258(6)	0.0249(6)	0.0251(6)	0.0249(6)
O2	x	0.9794(5)	0.9810(3)	0.9796(3)	0.9799(3)	0.9797(3)
18g	U_{iso}	0.0061(10)	0.0258(6)	0.0249(6)	0.0251(6)	0.0249(6)
y = x–2/3						
z = 1/6						
O3	x	0.1218(2)	0.1212(1)	0.1206(2)	0.1204(1)	0.1205(1)
18h	z	0.1353(5)	0.1333(3)	0.1320(2)	0.1321(2)	0.1321(2)
y = 2x	U_{iso}	0.0061(10)	0.0258(6)	0.0249(6)	0.0251(6)	0.0249(6)
O4	y	0.2624(4)	0.2645(3)	0.2627(3)	0.2631(2)	0.2628(3)
18f	U_{iso}	0.0061(10)	0.0258(6)	0.0249(6)	0.0251(6)	0.0249(6)
x = z = 0						
A (Cu^{2+})	z				0.147(5)	0.146754[a]
6c	U_{iso}				0.11(5)	0.01(3)[b]

x = *y* = 0	*occ*				0.025(3) / 0.15(2)	0.008(2) / 0.05(1)
A’ (Cu^{2+})	*x*			0.040(3)		0.039027[a]
18*h*	*z*			0.150(4)		0.149914[a]
y = 2*x*	U_{iso}			0.10(5)		0.01(3)[b]
	occ			0.013(1) / 0.22(2)		0.008(1) / 0.14(2)
B (Cu^{2+})	*x*			0.997(3)	0.996(4)	0.997(3)
36*i*	*y*			0.413(3)	0.413(3)	0.412(3)
	z			0.069(2)	0.068(3)	0.068(2)
	U_{iso}			0.16(3)	0.16(3)	0.16(3)
	occ			0.022(1) / 0.80(4)	0.021(1) / 0.76(4)	0.022(1) / 0.79(4)
Reflections		1099	1101	1101	1101	1101
a (Å)		13.5799(2)	13.5693(1)	13.5692(2)	13.5693(1)	13.5692(2)
c (Å)		14.7472(3)	14.8156(2)	14.8156(3)	14.8156(2)	14.8156(3)
V ($Å^3$)		2355.22(5)	2362.46(4)	2362.44(7)	2362.45(3)	2362.44(7)
wRp (%)		3.42	2.97	2.67	2.68	2.67
Rp (%)		2.12	2.19	1.99	1.98	1.98
χ^2		1.08	1.16	1.04	1.05	1.04
S^2 (MEM)		2.14	2.29	2.03	2.01	2.03
χ^2_{opt} (MEM)		1.51	0.66	1.6	1.5	1.6

[a]Not refined. [b]Refined as a single parameter.

Table S2 Selected distances [Å] and angles [°].

Distances/Angles	H	Cu			
		Framework	A’ + B	A + B	A + A’ + B
T–O1	1.639(3)	1.622(2)	1.613(2)	1.611(2)	1.612(2)
T–O2	1.605(7)	1.610(4)	1.603(4)	1.605(4)	1.604(4)
T–O3	1.586(6)	1.611(3)	1.614(3)	1.613(3)	1.613(3)
T–O4	1.612(3)	1.611(2)	1.600(2)	1.604(2)	1.601(2)

<T–O>	1.610(10)	1.613(6)	1.608(6)	1.608(6)	1.608(6)
A–O1				2.362(13)	2.363(3)
A'–O1			2.075(56)		2.081(2)
A'–O3			1.925(82)		1.934(4)
B–O2			1.917(36)	1.929(45)	1.919(36)
O4–T–O3	114.2(3)	112.7(2)	111.6(1)	111.7(1)	111.7(1)
O4–T–O2	108.1(1)	108.08(7)	109.30(7)	108.94(7)	109.17(7)
O4–T–O1	108.3(2)	109.6(1)	109.3(1)	109.4(1)	109.3(1)
O3–T–O2	107.9(3)	106.7(2)	107.5(2)	107.5(2)	107.5(1)
O3–T–O1	110.8(3)	108.9(2)	108.9(2)	108.8(2)	108.9(2)
O2–T–O1	107.3(2)	110.9(1)	110.2(1)	110.5(1)	110.3(1)
T–O1–T	151.7(2)	148.8(1)	149.2(2)	149.2(1)	149.3(1)
T–O2–T	151.5(1)	148.14(8)	148.92(7)	148.56(8)	148.79(7)
T–O3–T	144.9(4)	146.4(2)	147.9(2)	147.9(2)	147.8(2)
T–O4–T	146.6(1)	144.92(6)	146.67(6)	146.17(6)	146.50(6)
O1–A'–O3			81.6(26)		81.5(1)

S3. MEM

The unit cell for all samples was divided into 90x90x120 pixels in the *a*, *b*, and *c* direction, respectively, giving a grid size of ~0.15 Å. The relatively large grid size is chosen to lower the calculation time. A test calculation at high resolution with a grid size of ~0.05 Å showed no change in the conclusions drawn from the relatively low resolution calculations.

It is assumed the experimental errors on the absolute scale observed structure factors, $\left|F_{obs}(\vec{H}_i)\right|$, are random with a Gaussian distribution, giving the goodness-of-fit/stopping criteria:

$$\chi^2 = \frac{1}{N_F}\sum_{i=1}^{N_F}\left(\frac{\left|F_{obs}(\vec{H}_i) - F_{MEM}(\vec{H}_i)\right|}{\sigma\left(F_{obs}(\vec{H}_i)\right)}\right)^2 \qquad (3.2)$$

N_F is the number of observed structure factors $F_{obs}(\vec{H})$, $F_{MEM}(\vec{H})$ denotes the structure factors calculated for the current estimate of the EDD, and $\sigma(F_{obs}(\vec{H}))$ is the standard deviation of $F_{obs}(\vec{H})$. Normally the criterion for MEM convergence is $\chi^2 = 1$, but the actual optimal χ^2 value depends on the Rietveld refinement program used and the quality of the data. The reason is the standard deviation determination, which differs with each program. To ensure the optimum χ^2 value, and the optimal

MEM EDD, Residual Density Analysis (RDA) has been performed according to Bindzus and Iversen (2012), (Meindl & Henn, 2008).

S3.1. Residual Density Analysis

The fractal dimension distributions for different χ^2 aims of MEM electron density distributions (EDDs) of H-CHA data are shown in Figure S9. Coefficients of determination, R^2, from these fits are shown in Figure S10, giving χ^2_{opt} = 1.51.

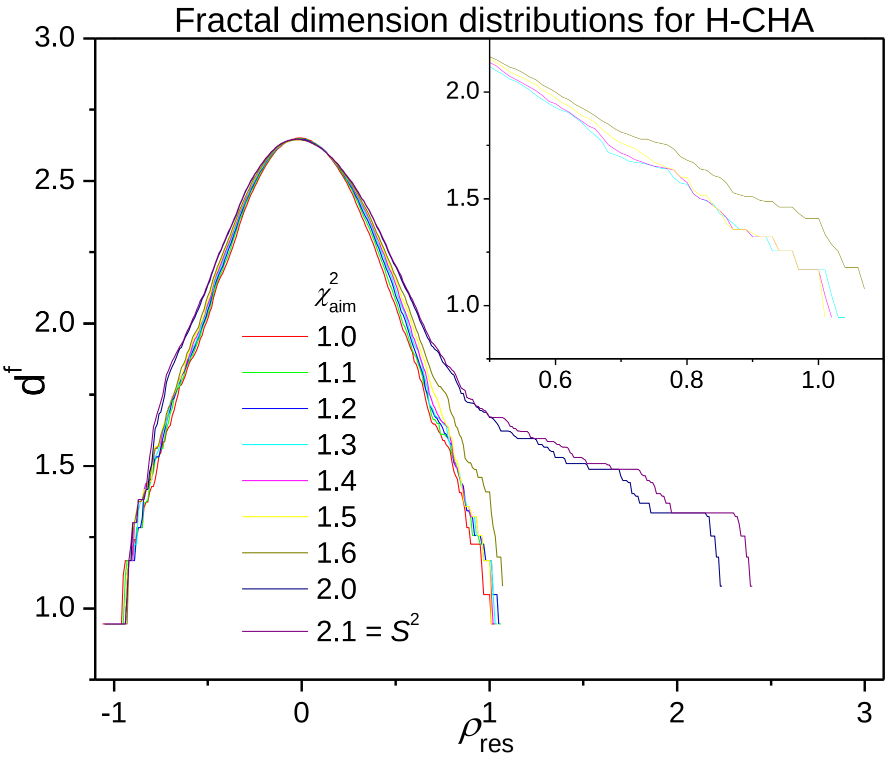

Figure S9 Fractal dimension distributions calculated from MEM residual densities, ρ_{res}, computed by the inverse Fourier transformation of the difference $F_{obs}(\vec{H})$-$F_{MEM}(\vec{H})$. S^2 is the max. goodness-of-fit defined by the prior.

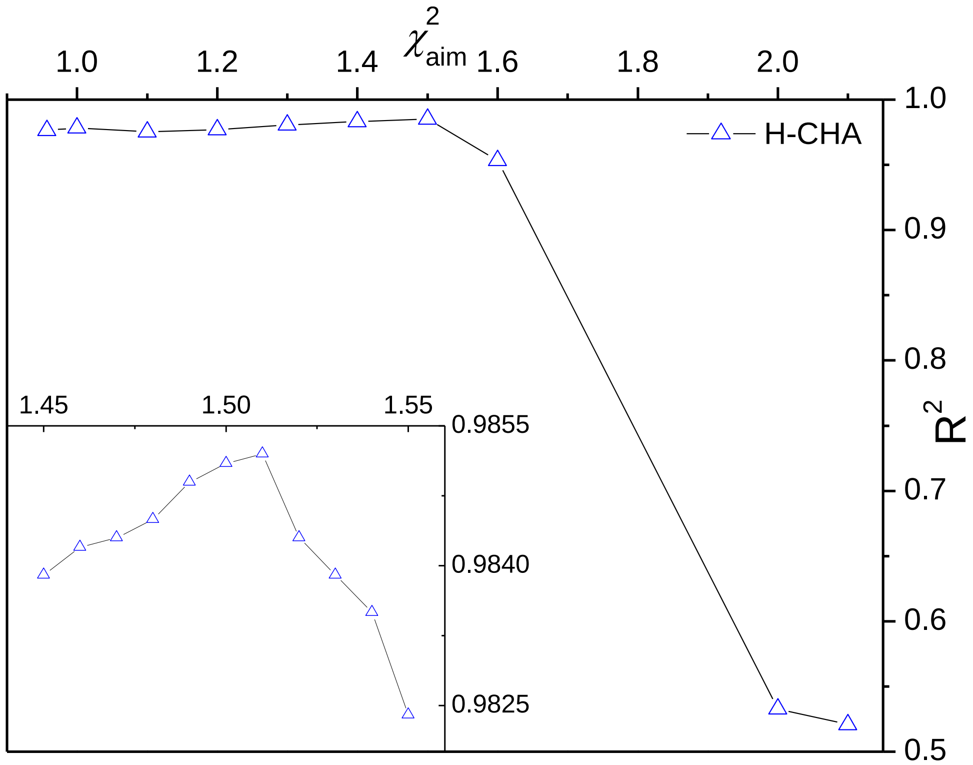

Figure S10 Coefficients of determination, R^2, obtained by fitting the fractal dimension distributions shown in Figure S9 to a parabolic function, $f(x) = c_1x^2+c_2$. In the inset further R^2 are shown, based on $d^f(\rho_{res})$'s calculated around $\chi^2_{aim} = 1.50$.

S3.2. MEM EDDs

To be able to compare the MEM EDDs, the isosurface level of electron density (ED) for all MEM EDD figures is set to 0.55 e/Å^3. The MEM EDD of H-CHA is shown in Figure S11 and Figure S12. It is clear that no extra ED is found outside of the framework. The small densities found in the second EDD in Figure S11 belong to the framework of the next unit cell. The MEM EDD shows that the structural model for H-CHA is complete. It also shows how effective the dehydration technique is. Since no ED is found outside the framework, any ED found outside the framework of the metal loaded CHA zeolites must be due to guest species.

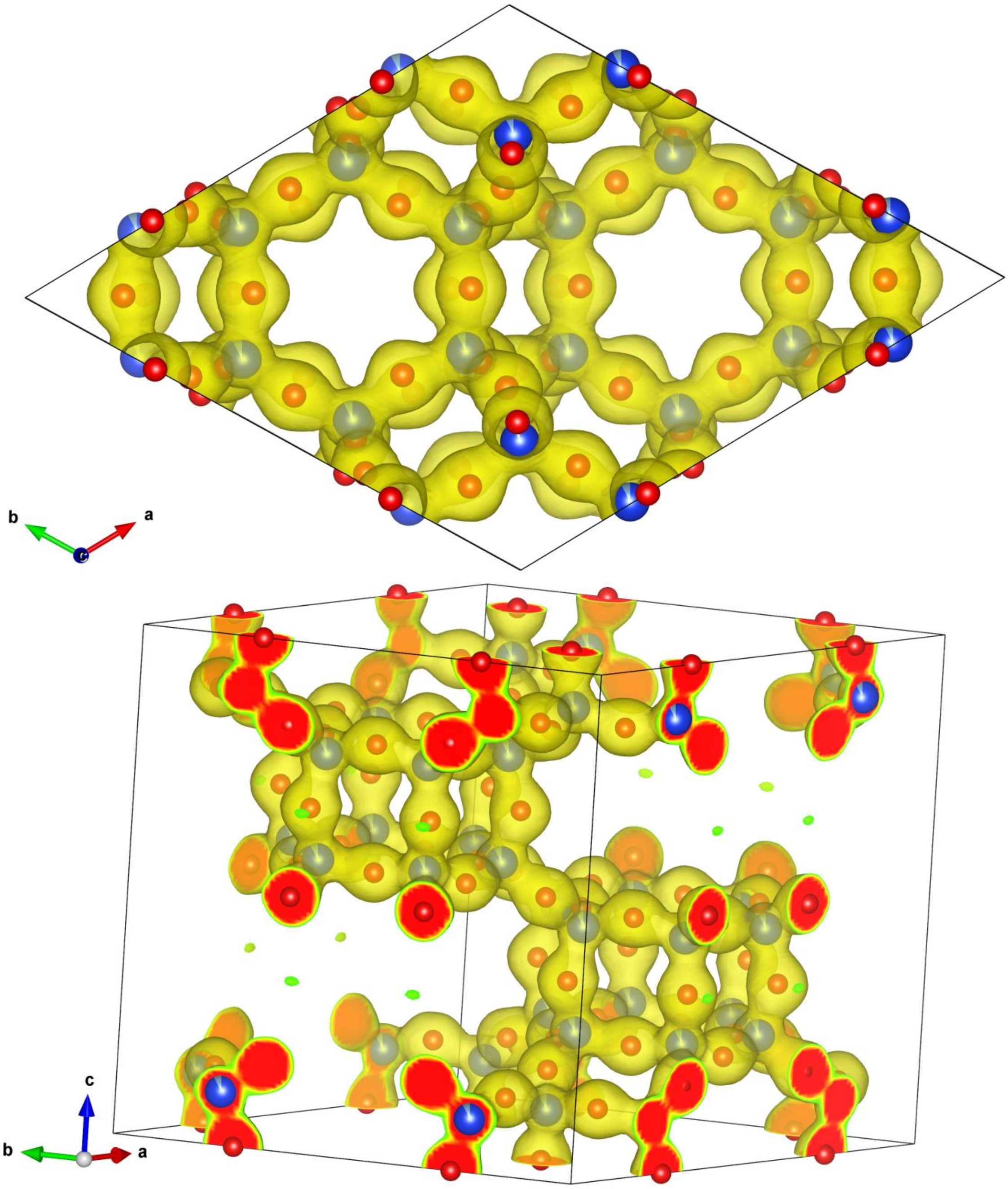

Figure S11MEM EDDs of H-CHA with the framework as a prior. The density max is ρ_{max} = 290 e/Å^3.

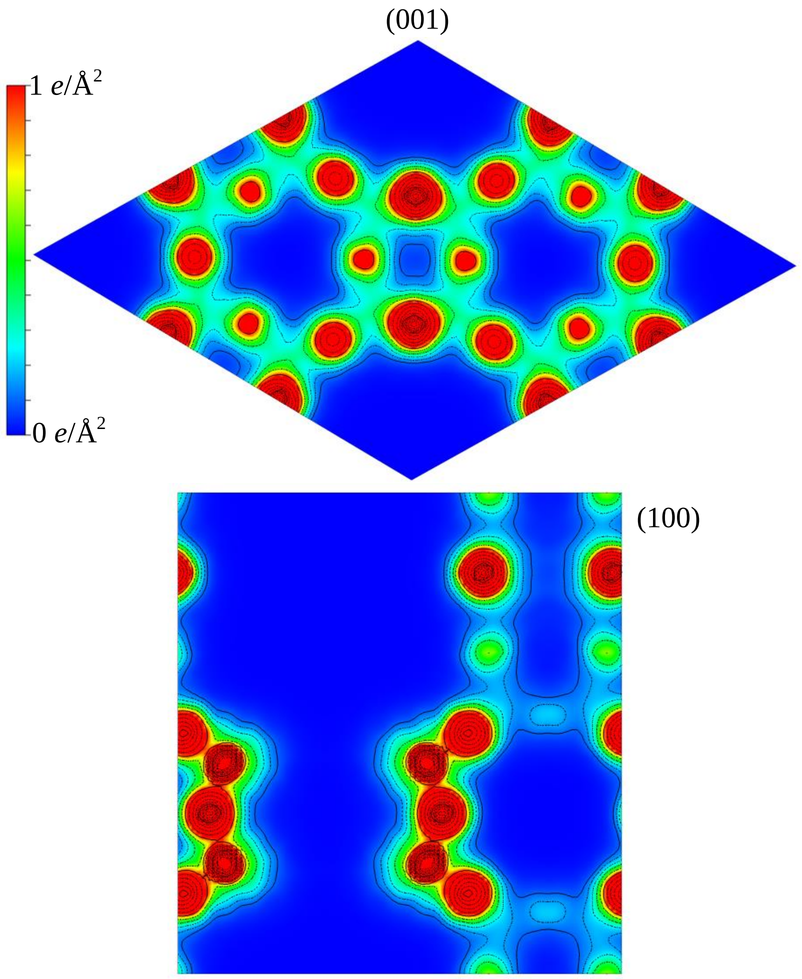

Figure S12MEM EDD contour maps of H-CHA with the framework as a prior. The (001) and (100) planes at z = 1/2 and x = 2/3, respectively, are shown. The contour map scale goes from 0 e/Å^2 (blue) to 1 e/Å^2 (red), the contour lines are logarithmic by $10^{N/5}$, where N = [-1;2].

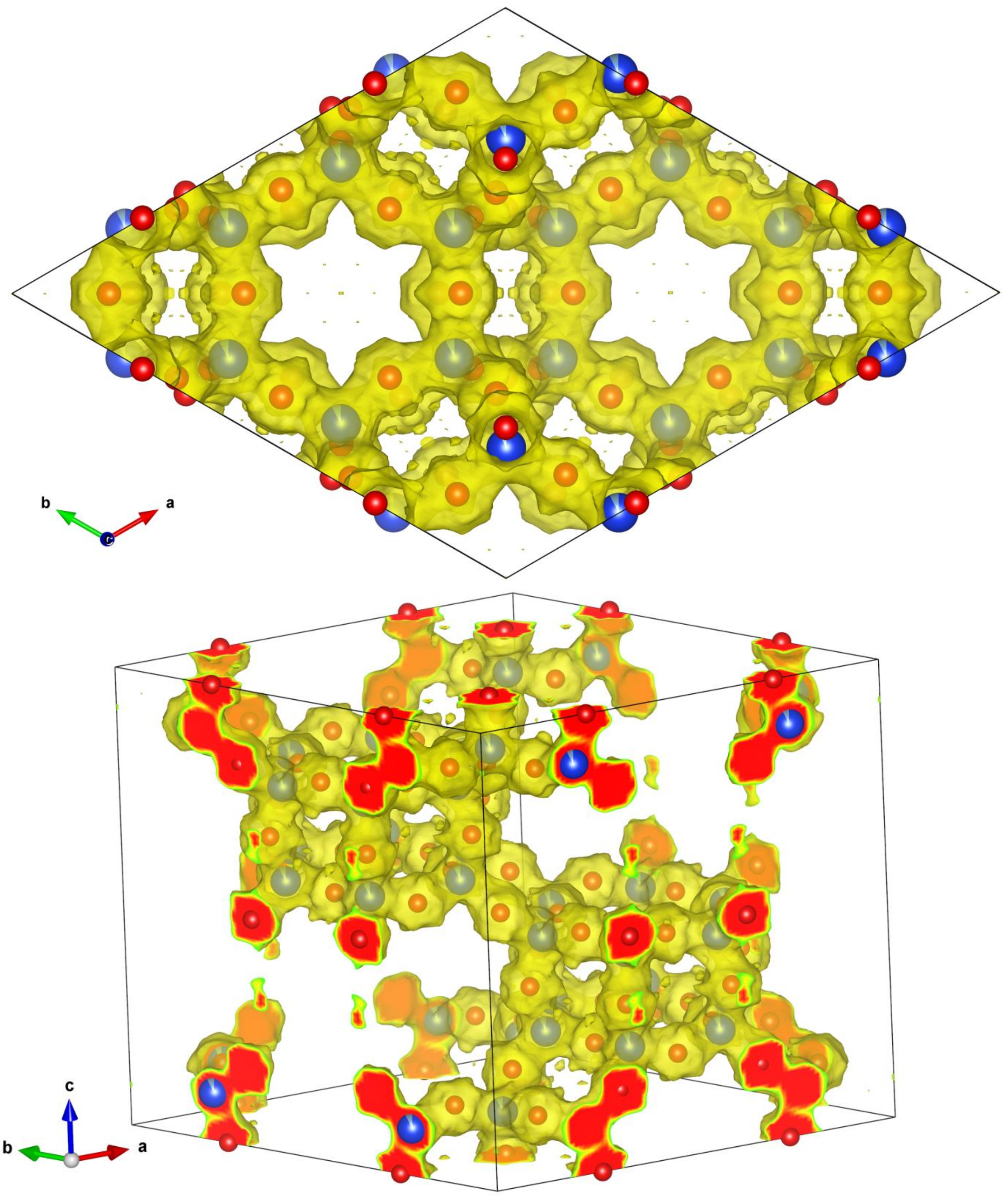

Figure S13MEM EDDs of Cu-CHA with the framework as a prior. Notice the small specks of EDD distributed in the cages. The density max is $\rho_{\mathrm{max}} = 85\ e/\text{Å}^3$.

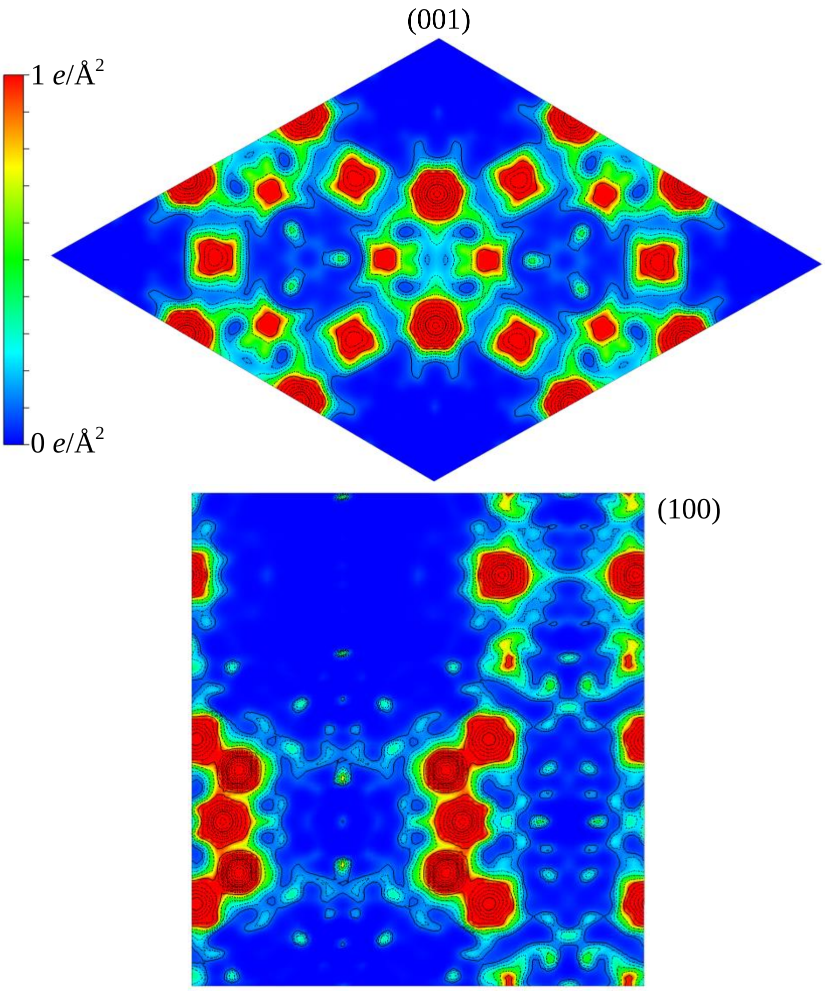

Figure S14MEM EDD contour maps of Cu-CHA with the framework as a prior. The (001) and (100) planes at $z = 1/2$ and $x = 2/3$, respectively, are shown. The contour map scale goes from 0 e/Å^2 (blue) to 1 e/Å^2 (red), the contour lines are logarithmic by $10^{N/5}$, where N = [-1;2].

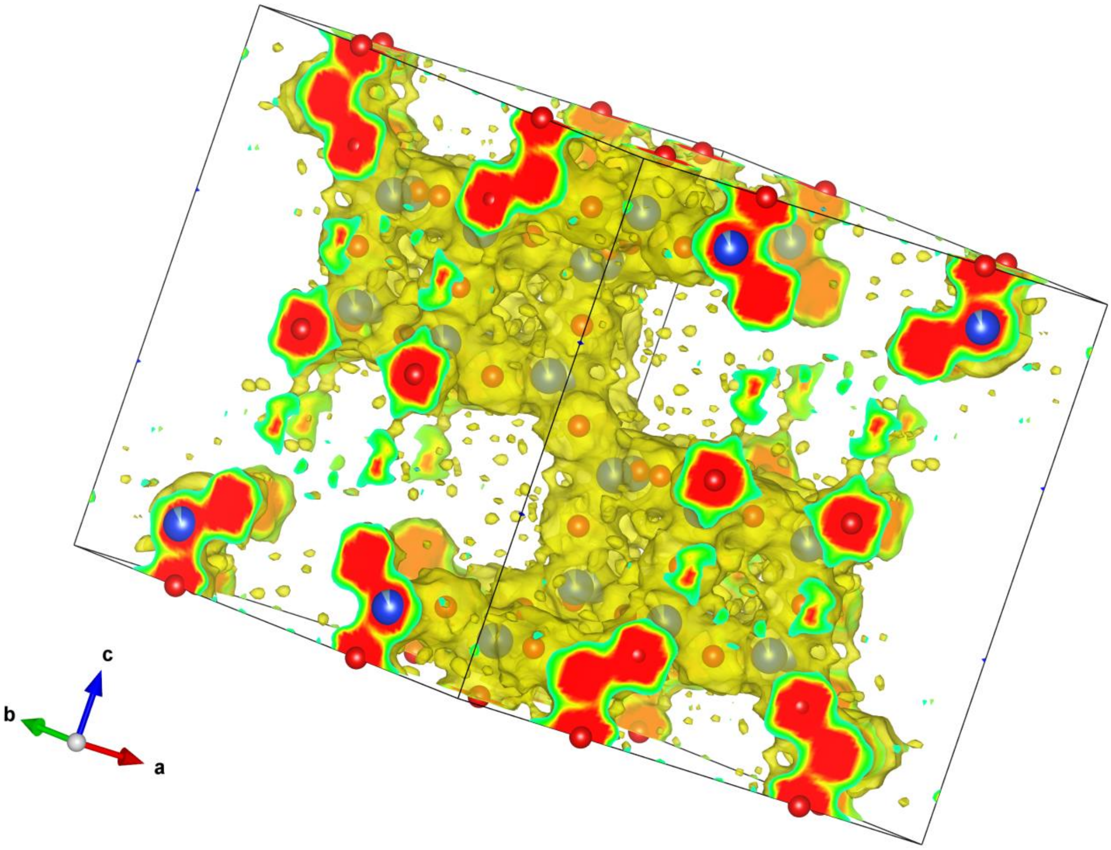

Figure S15MEM EDD of Cu-CHA with the framework as a prior. The isosurface level has been lowered to 0.3 e/Å^3. The density max is ρ_{max} = 85 e/Å^3.

The MEM EDD of Cu-CHA using the framework-only model displays a lot of non-framework features in the ED, see Figure S13 and Figure S14. It is especially visible when the isosurface level is lowered, see Figure S15.

In the following figures the MEM EDDs of Cu-CHA using the three different Cu^{2+} ion models are shown. Below (Figure S16) is also a close up of a 6-ring in the model incl. sites A, A', and B, where the isosurface level has been lowered in order to see the A and A' sites.

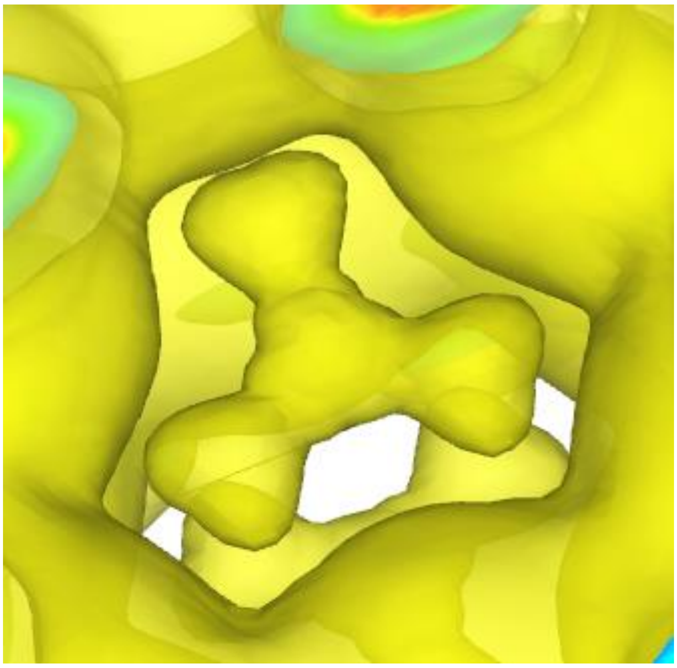

Figure S16MEM EDD close up of a 6-ring. Taken from the EDD of Cu-CHA with the framework and Cu^{2+} sites A, A’, and B. The isosurface level has been lowered.

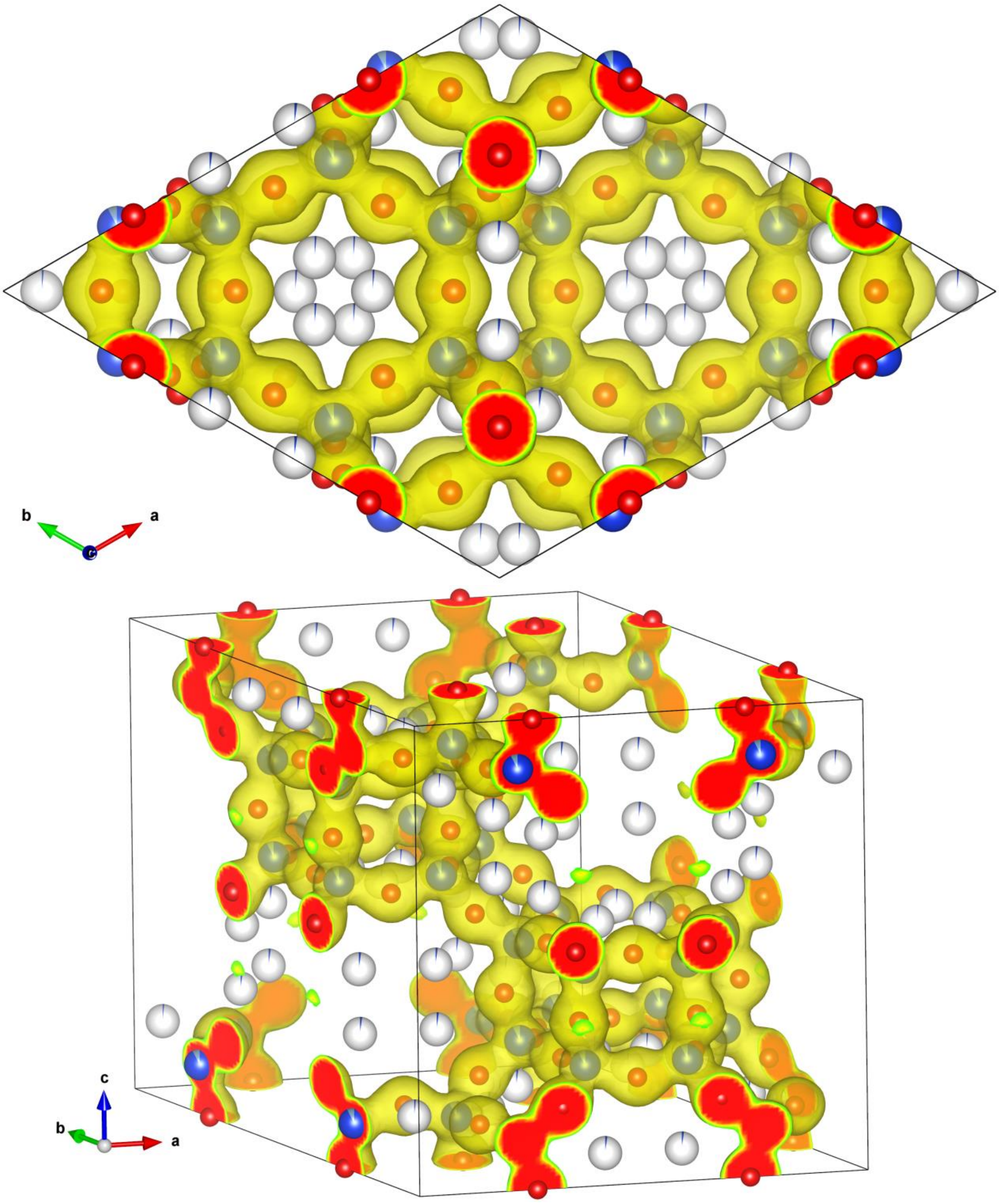

Figure S17MEM EDDs of Cu-CHA with the framework and Cu^{2+} sites A' and B as a prior. The density max is ρ_{max} = 88 e/Å^3.

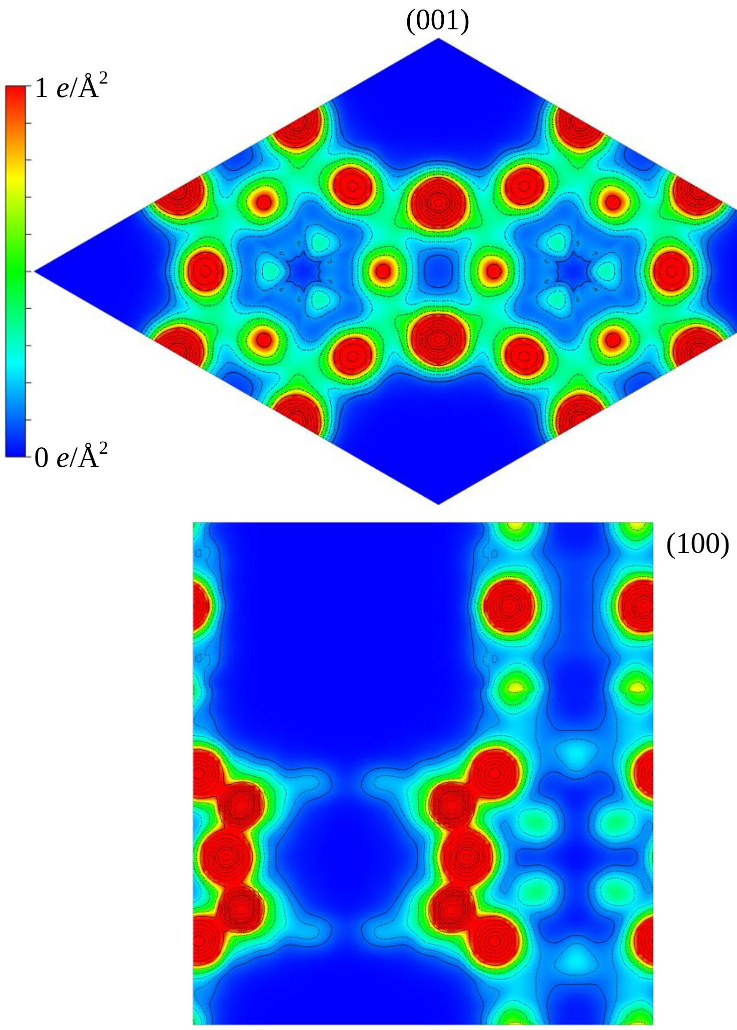

Figure S18MEM EDD contour maps of Cu-CHA with the framework and Cu^{2+} sites A' and B as a prior. The (001) and (100) planes at z = 1/2 and x = 2/3, respectively, are shown. The contour map scale goes from 0 e/Å^2 (blue) to 1 e/Å^2 (red), the contour lines are logarithmic by $10^{N/5}$, where N = [-1;2].

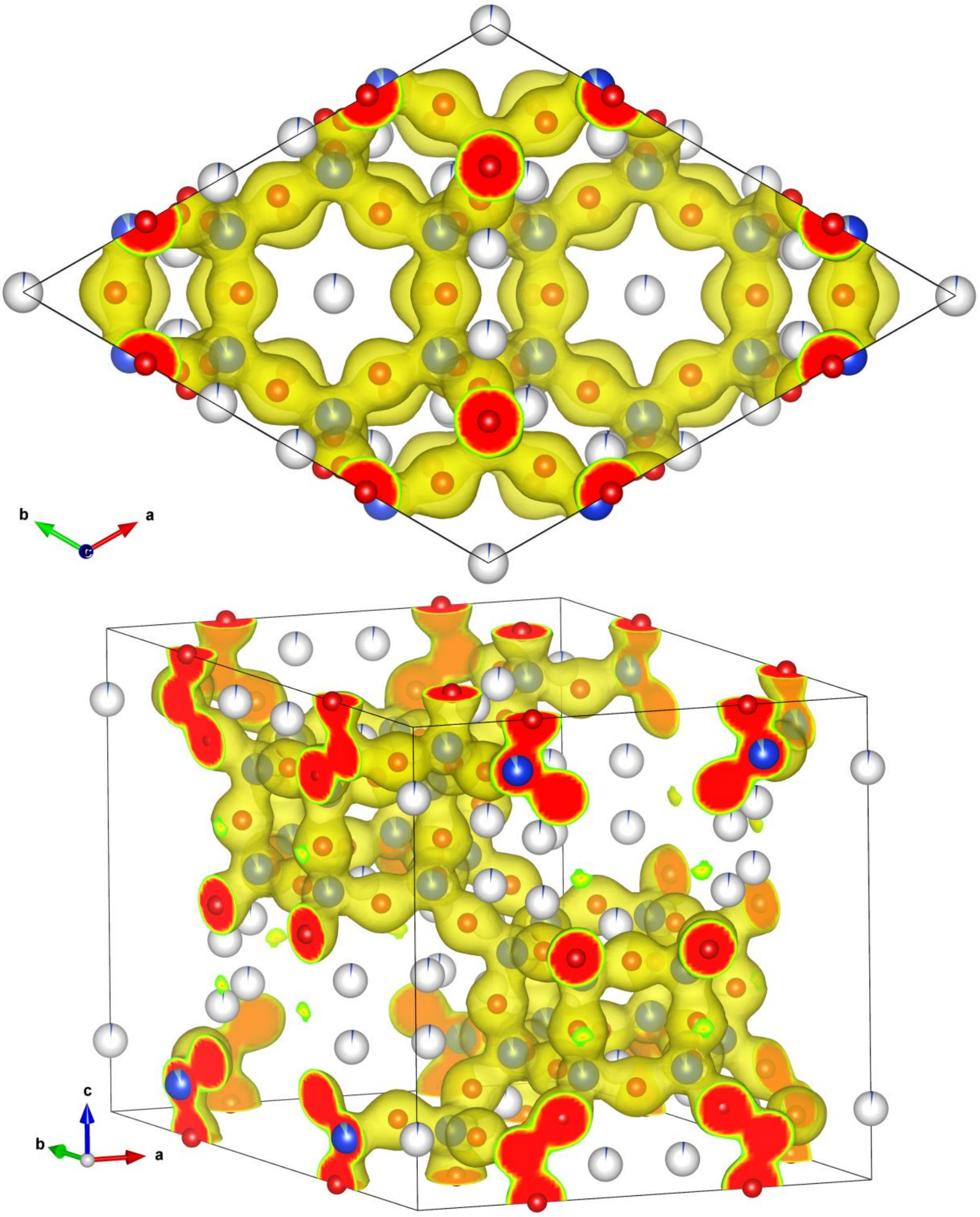

Figure S19MEM EDDs of Cu-CHA with the framework and Cu^{2+} sites A and B as a prior. The density max is ρ_{max} = 88 e/Å^3.

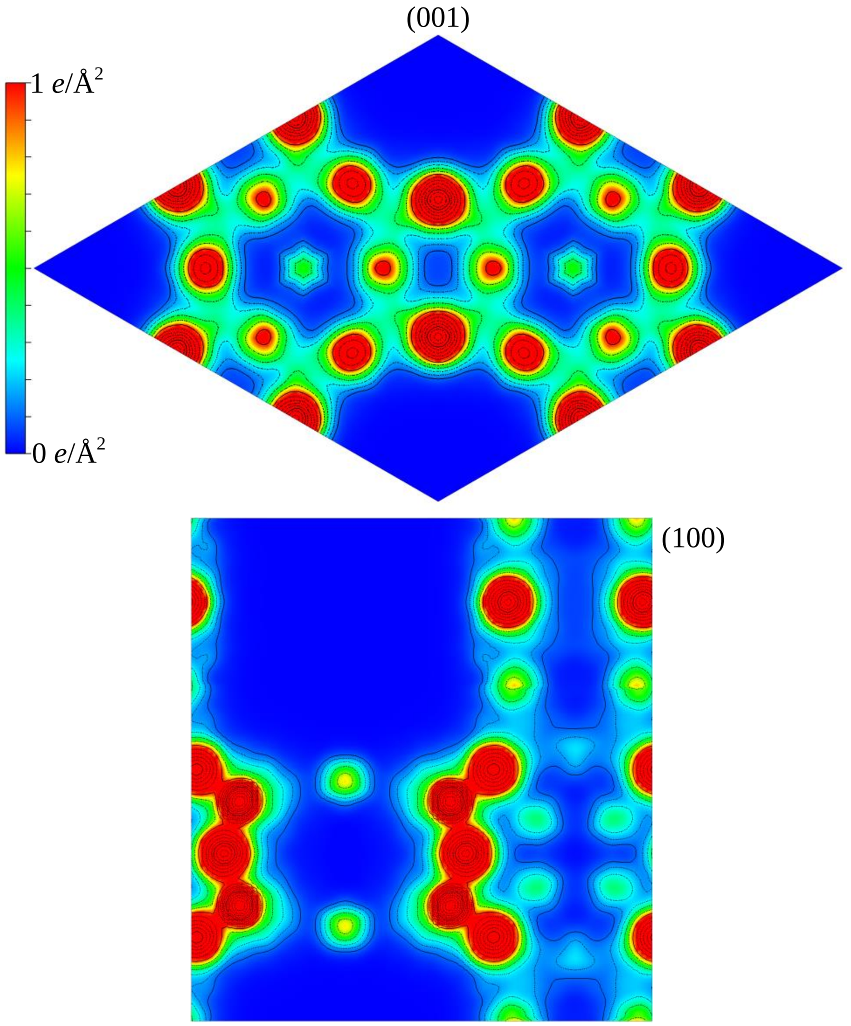

Figure S20MEM EDD contour maps of Cu-CHA with the framework and Cu^{2+} sites A and B as a prior. The (001) and (100) planes at z = 1/2 and x = 2/3, respectively, are shown. The contour map scale goes from 0 e/Å^2 (blue) to 1 e/Å^2 (red), the contour lines are logarithmic by $10^{N/5}$, where N = [-1;2].

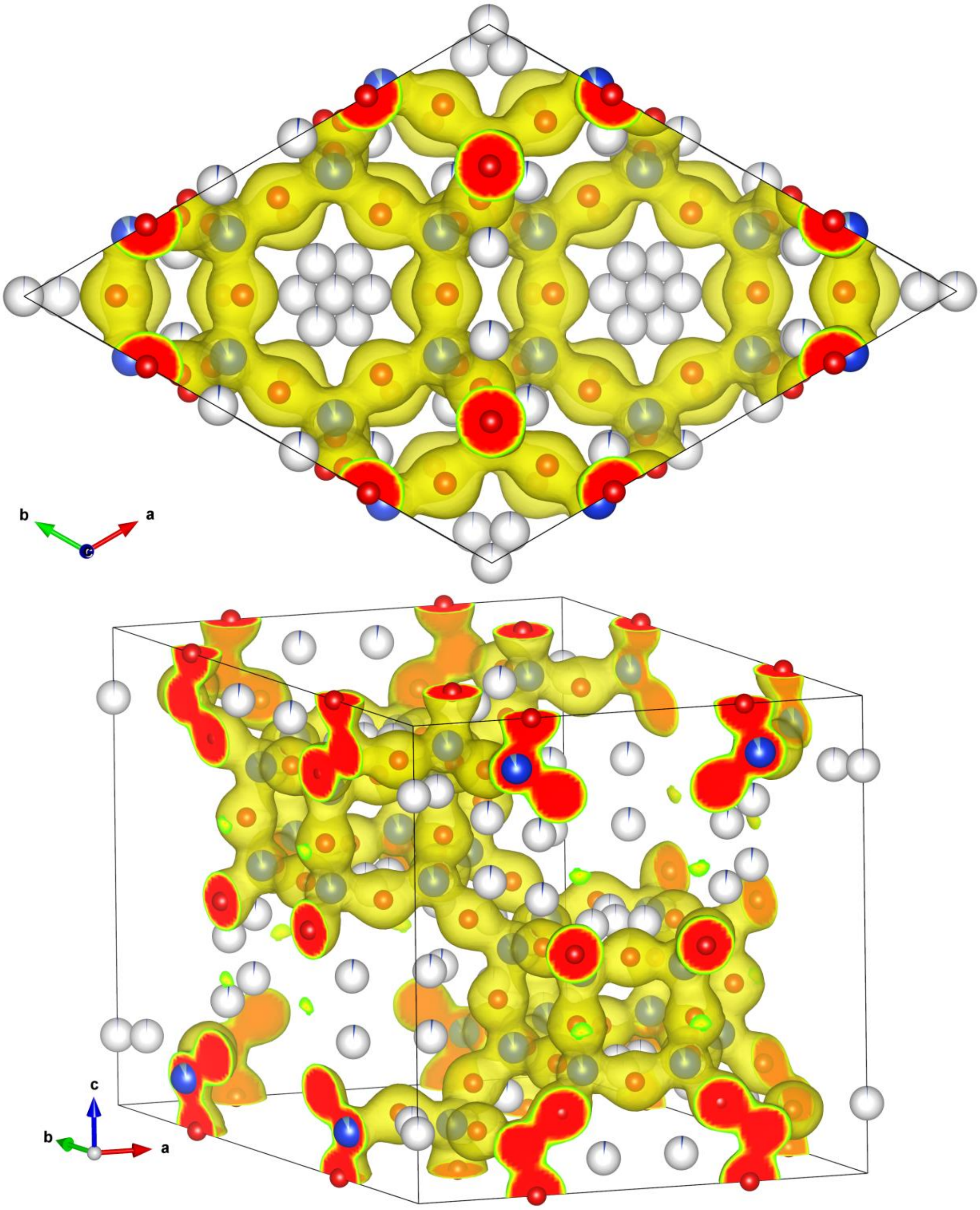

Figure S21MEM EDDs of Cu-CHA with the framework and Cu^{2+} sites A, A', and B as a prior. The density max is ρ_{max} = 88 e/Å^3.

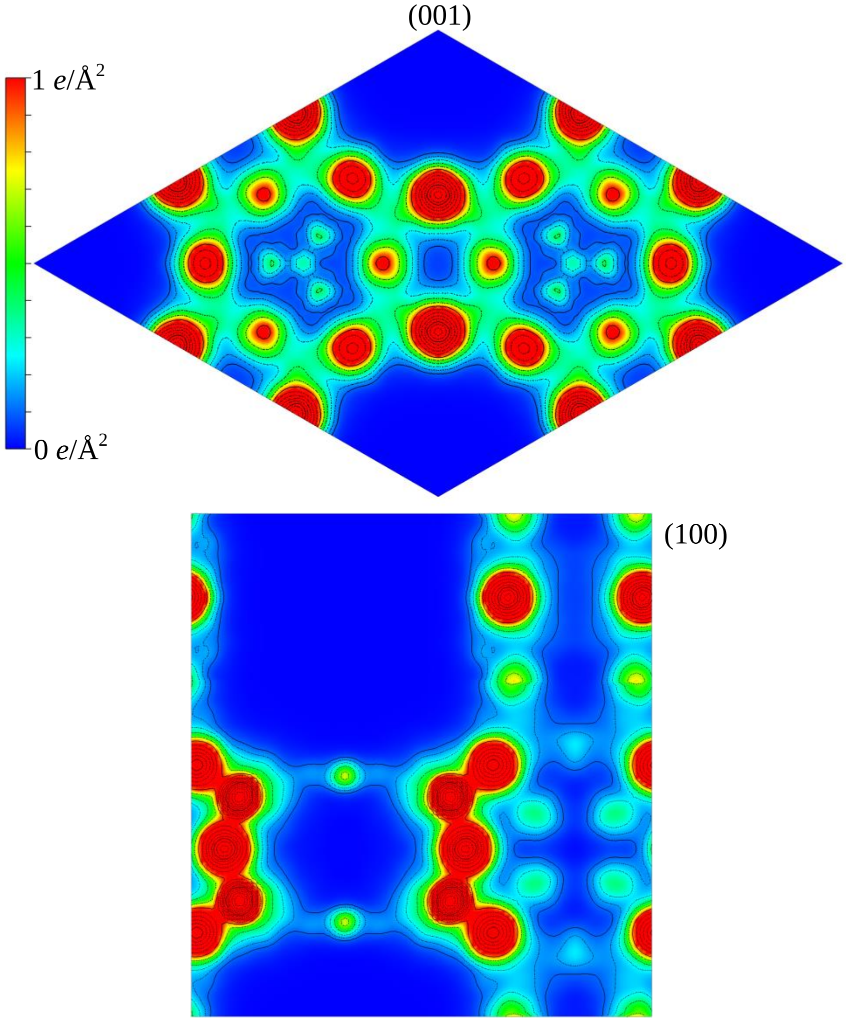

Figure S22 MEM EDD contour maps of Cu-CHA with the framework and Cu^{2+} sites A, A', and B as a prior. The (001) and (100) planes at *z* = 1/2 and *x* = 2/3, respectively, are shown. The contour map scale goes from 0 *e*/Å^2 (blue) to 1 *e*/Å^2 (red), the contour lines are logarithmic by $10^{N/5}$, where *N* = [-1;2].

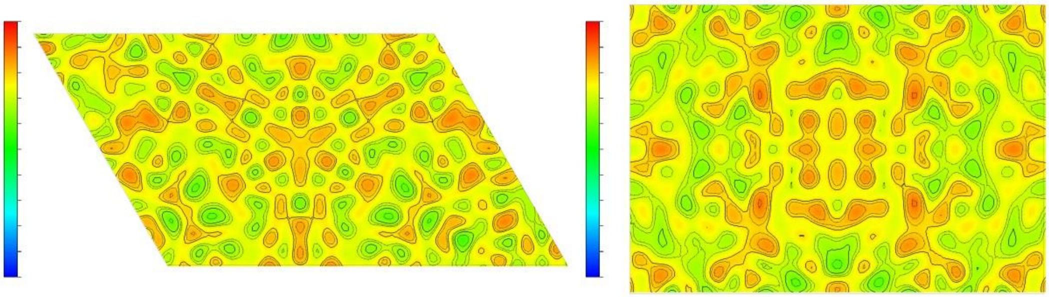

Figure S23 Standard difference Fourier maps obtained using only the framework atoms in the Rietveld model. As can be see the maps are noisy and cannot be used to precisely locate the Cu atoms.

S4. DFT

All density functional theory (DFT) calculations were performed using a real space grid-based projector augmented wave method (GPAW) (Mortensen *et al.*, 2005, Enkovaara *et al.*, 2010) interfaced using the atomic simulation environment (ASE) (Bahn & Jacobsen, 2002). For all calculations, periodic boundary conditions were used and the RPBE functional applied. Additionally, for all calculations only the Γ-point of the Brillouin zone was sampled using 0.1 eV Fermi smearing, which was found sufficient based on convergence tests.

Initially the CHA unit cell with the $R\bar{3}m$ space group was obtained from the IZA structure database (IZA-SC, 2007) and was optimized using a 0.15 Å grid spacing, where the number of grid points was kept constant. First, the purely siliceous unit cell was optimized based on the volume, whereupon both *a* and *c* parameters were varied independently. In all cases all atomic positions in the unit cell were allowed to relax and a force threshold of 0.03 eV/Å was applied. The obtained unit cell based on this optimization routine with lattice constants a = 13.87 Å and c = 15.12 Å was used as the basis for all further calculations.

In the optimized unit cell Al was isomorphously substituted with Si (all T-sites are initially equivalent by symmetry, so the choice of Si when only one Al is introduced is irrelevant). Cu as well as O and H, when needed, were also introduced and due to the presence of Cu, the calculations were done applying spin polarization. The grid spacing was increased to 0.20 Å in order to increase calculation speed. Besides this, all parameters remained the same as stated for the optimization of the unit cell.

In the case of replacing only one Si with an Al atom and introducing a single Cu atom, the Cu atom can be coordinated to any of the oxygen atoms surrounding the Al. Thus, a calculation was made for each of the possible Cu coordinations, where the atom positions were allowed to relax to an energy minimum. This procedure was followed for all cases after introduction of the Cu moieties in the unit cell.

When a single Al and a single Cu was introduced only two stable locations could be found, which corresponds to the Cu in the plane of the 6R and in the plane of the 8R. However, the site in the 6R was found to be 0.26 eV more favorable than the site in the 8R. The magnetic moment converged to a value of zero, indicating that this type of Cu species corresponds to the Cu ion in oxidation state +1, and is therefore considered less relevant for this specific study.

Similarly, Cu with an OH-ligand was also introduced, which has recently been suggested from spectroscopic investigations as a relevant Cu species in dehydrated zeolites (Giordanino *et al.*, 2013). This leads to a Cu complex where Cu is in oxidation state +2, as also witnessed by the converged non-zero magnetic moment. Again all possible initial locations were attempted, but after relaxation of the atom positions only two stable sites were found. These correspond to the $[Cu(OH)]^+$ complex located in the plane of the 8R and one where the $[Cu(OH)]^+$ complex is moved slightly out of the 6R and into the large CHA cage in between the 6R and the 8R. In this case the most stable configuration is the $[Cu(OH)]^+$ complex in the 8R, being 0.08 eV more favored than the other.

When two Al atoms are isomorphously substituted for Si into the unit cell many options exist for the location of the second Al atom, which is expected to obey Löwensteins rule (no Al-O-Al bridges). All options of locating the second Al atom as a next-nearest-neighboring atom were attempted in combination with the various possibilities of locating a Cu atom coordinated to the O-atoms surrounding the Al. After relaxation of the atomic positions, two cases stood out being most stable (by more than 0.5 eV than the second most stable configuration). These are the configurations where both Al atoms are located in a 6R; either diagonally across the 6R or separated by a single Si T-atom. Again the +2 oxidation state of Cu was confirmed by the non-zero magnetic moment.

The most stable configurations found as mentioned in the text above is given in Figure 2 in the main text. Furthermore, chosen distances and angles are given in Table S3.

Table S3 Selected distances [Å] and angles [°] for DFT calculations.

	Distances/Angles
A–O1	2.005
A'–O1	2.104
A'–O3	1.964
B–O2	1.796
O1–A'–O3	77.2

S5. References

Bahn, S. R. & Jacobsen, K. W. (2002). *Computing in Science & Engineering* **4**, 56-66.
Berar, J.-F. & Baldinozzi, G. (1993). *Journal of Applied Crystallography* **26**, 128-129.
Bindzus, N. & Iversen, B. B. (2012). *Acta Crystallogr. Sect. A* **68**, 750-762.
Diaz-Cabanas, M.-J. & A. Barrett, P. (1998). *Chem. Commun.* **17**, 1881-1882.
Eilertsen, E. A., Arstad, B., Svelle, S. & Lillerud, K. P. (2012). *Micropor. Mesopor. Mat.* **153**, 94-99.
Enkovaara, J., Rostgaard, C., Mortensen, J. J., Chen, J., Dułak, M., Ferrighi, L., Gavnholt, J., Glinsvad, C., Haikola, V., Hansen, H. A., Kristoffersen, H. H., Kuisma, M., Larsen, A. H., Lehtovaara, L., Ljungberg, M., Lopez-Acevedo, O., Moses, P. G., Ojanen, J., Olsen, T., Petzold, V., Romero, N. A., Stausholm-Møller, J., Strange, M., Tritsaris, G. A., Vanin, M., Walter, M., Hammer, B., Häkkinen, H., Madsen, G. K. H., Nieminen, R. M., Nørskov, J. K., Puska, M., Rantala, T. T., Schiøtz, J., Thygesen, K. S. & Jacobsen, K. W. (2010). *Journal of Physics: Condensed Matter* **22**, 253202.
Giordanino, F., Vennestrom, P. N. R., Lundegaard, L. F., Stappen, F. N., Mossin, S., Beato, P., Bordiga, S. & Lamberti, C. (2013). *Dalton T.* **42**, 12741-12761.
IZA-SC (2007). *Database of zeolite structures: Http://www.Iza-structure.Org/databases/*.
Meindl, K. & Henn, J. (2008). *Acta Crystallogr. Sect. A* **64**, 404-418.
Mortensen, J. J., Hansen, L. B. & Jacobsen, K. W. (2005). *Phys Rev B* **71**, 035109.
